# Combined multi-omics and physiological approaches to elucidate drought-response mechanisms of durum wheat

**DOI:** 10.3389/fpls.2025.1540179

**Published:** 2025-05-15

**Authors:** Osvin Arriagada, Claudio Meneses, Romina Pedreschi, Gerardo Núñez-Lillo, Carlos Maureira, Samantha Reveco, Valentina Villarroel, Úrsula Steinfort, Francisco Albornoz, Patricia Cabas-Lühmann, Manuela Silva, Iván Matus, Andrés R. Schwember

**Affiliations:** ^1^ Centro de Estudios en Alimentos Procesados (CEAP), Talca, Chile; ^2^ Facultad de Ciencias Biológicas, Pontificia Universidad Católica de Chile, Santiago, Chile; ^3^ Escuela de Agronomía, Facultad de Ciencias Agronómicas y de los Alimentos, Pontificia Universidad Católica de Valparaíso, Quillota, Chile; ^4^ Millennium Institute Center for Genome Regulation, Santiago, Chile; ^5^ Departamento de Ciencias Vegetales, Facultad de Agronomía y Sistemas Naturales, Pontificia Universidad Católica de Chile, Santiago, Chile; ^6^ Linking Landscape, Environment, Agriculture and Food Research Center (LEAF), Associate Laboratory TERRA, Instituto Superior de Agronomia, Universidade de Lisboa, Lisboa, Portugal; ^7^ Instituto de Investigaciones Agropecuarias (INIA)-Quilamapu, Programa de Mejoramiento Genético de trigo, Chillán, Chile

**Keywords:** *Triticum durum* L., proline, WRKY, QTL, GWAS, RNA-seq

## Abstract

**Introduction:**

Durum wheat is the most important cereal in the Mediterranean regions, where drought negatively affects grain yield. Therefore, our objective was to perform a multi-omics and integration analysis in conjunction with physiological trials to improve our understanding of drought tolerance mechanisms of durum wheat.

**Methods:**

Genome-wide association study (GWAS) for yield components was performed on a panel of 225 elite durum wheat genotypes evaluated in eight sites under irrigated and rainfed conditions. Based on physiological parameters (net photosynthesis, intracellular CO2 content, transpiration and stomatal conductance) and grain yield, contrasting genotypes (susceptible and tolerant) to drought were identified. A transcriptomic (RNA-seq), metabolomic and integration analyses were performed to identify genes and metabolites associated with tolerance in durum wheat.

**Results:**

Nine marker-trait associations were detected across 8 environments, and they were grouped into three QTL clusters (QTL_2A_TGW/GPS.1, QTL_2A_TGW/GPS.2, and QTL_2B_TGW/GPS.1), explaining between 5.15% and 14.29% of the phenotypic variation. One drought tolerant (QUC 3678-2016) and one susceptible (BRESCIA) genotype were identified based on physiological parameters. RNA-seq analysis showed that the genes regulated were mainly enriched in processes such as response to salicylic acid, plant organ senescence, synthesis of secondary metabolites, and immune response. Metabolic analysis showed that drought increased the contents of amino acids, sugars, and organic acids. The integration analysis identified 30 genes and six metabolites in the root and 30 genes and 10 metabolites in leaves as the primary variables in the drought-tolerant genotype, in which L-Proline was an important metabolite that allowed differentiating those two contrasting genotypes. A WRKY transcription factor was also positioned on the stable QTL QTN_2A_TGW/GPS.1 associated with the GENE-1342_238 SNP marker.

**Discussion:**

These results open an opportunity to use new biomarkers in durum wheat breeding programs to develop resilient and high-yielding cultivars and ensure food security under water deficit conditions.

## 1 Introduction

Durum wheat (*Triticum turgidum* L. ssp. *durum*; 2n = 4 × = 28, AABB) is an essential crop in the world with a cultivated area of 17 million ha and a production of 33.6 million t in 2020 (Royo et al., 2021). This crop is the most important cereal in the Mediterranean region since it is deeply connected with the history and culinary traditions of this region (Martínez-Moreno et al., 2020). However, only half of the worldwide production is produced in this area (Zaïm et al., 2024). Among the main durum wheat-producing countries in the world are Spain, France, Italy, and Greece in southern Europe; Morocco, Algeria, and Tunisia in northern Africa; Turkey and Syria in southwest Asia; Canada, the United States, and Mexico in North America (Martínez-Moreno et al., 2020); and Argentina and Chile in South America (Colasuonno et al., 2019). Durum wheat plays a key role in the human diet because it is primarily used for making pasta and other semolina-based products, such as frike, couscous, bourghul, and unleavened bread, which are widely consumed in many countries of the world (Sharma et al., 2019), providing more than 20% of the nutritional demands for human consumption (Shewry and Hey, 2015).

Durum wheat is commonly grown in arid and semi-arid regions under rainfed conditions, where the water scarcity combined with high temperatures during grain-filling period significantly affects key plant metabolic pathways, causing grain yield losses of up to 50% to farmers (Dettori et al., 2017; Soriano et al., 2021). Additionally, global climate models predict a continued reduction in precipitation in the main durum wheat production areas (Schlaepfer et al., 2017; Daramola and Xu, 2022), which will significantly affect the production. Therefore, it is essential to study and understand the mechanisms that regulate drought tolerance in durum wheat to develop high-yielding varieties that are drought tolerance.

Plants have developed different physiological, biochemical, and molecular mechanisms to escape, tolerate, or avoid drought stress (Bashir et al., 2021; Dodd and Kudoyarova, 2021). Drought causes a reduction in leaf water potential (Ψ). The plant responds by closing its stomata, reducing the stomatal conductance, which affects transpiration and net photosynthesis rates and, ultimately, the grain yield (Gupta et al., 2020; Akhiyarova et al., 2023). The phytohormone abscisic acid (ABA) plays a key role in the regulation of stomatal closure under drought conditions (Hsu et al., 2021), where two signaling pathways in response to drought have been described, which are classified as ABA-dependent and ABA-independent (Yoshida et al., 2014). From a biochemical standpoint, drought signals induce stress-protective compounds with antioxidant activity, osmoprotectants, and secondary metabolites (Bashir et al., 2021). Finally, the drought response strategies are genetically controlled by numerous genes involved in osmolyte, redox, and hormonal metabolism (Singh PK. et al., 2019), such as dehydration-responsive element-binding factors (DREBs), basic leucine zipper (bZIP) proteins, MYBs, MYC, WRKY, NAC, sucrose non-fermenting1-related protein kinases (SnRKs) and SOS2-like protein kinases (CIPKs), among others (Joshi et al., 2016; Singh PK. et al., 2019; Bashir et al., 2021).

Genome-wide association studies (GWAS) have identified thousands of loci associated with complex traits in durum wheat (Sukumaran et al., 2018; Zaïm et al., 2024). However, the functional interpretation of GWAS results remains challenging due to large candidate regions and linkage disequilibrium (Knoch et al., 2024). Implementing and integrating different high-throughput omics techniques, including genomics, transcriptomics, proteomics, and metabolomics, have produced very valuable results in discovering specific genes, proteins, metabolites, and signaling processes in different tissues that are involved in the stress response in wheat (Shah et al., 2018; Goel et al., 2020; Da Ros et al., 2023; Le Roux et al., 2024). In addition, robust gene regulatory networks associated with drought stress have been constructed using multi-omics analysis in cereals (Baldoni et al., 2021), including wheat (Chen et al., 2025), which have had a major impact on accelerating crop improvement in the era of omics. However, multi-omics studies are still very limited for wheat. In fact, to our knowledge, multi-omics studies have only been performed in common wheat, opening the possibility of exploring and identify new markers, genes and metabolites that are associated with the response to drought in durum wheat. Therefore, this study aimed to perform physiological analyses and integrate different omics (genomics, transcriptomics, and metabolomics) approaches for a better understanding of the interaction between the different tolerance mechanisms involved in response to drought stress. These results are novel and valuable for developing resilient durum wheat cultivars, which are necessary to ensure production sustainability and global food security.

## 2 Materials and methods

All the experimental setup of this work is summarized in **Supplementary Figure S1**.

### 2.1 Genome-wide association approach

#### 2.1.1 Plant material and field management

A panel of 225 durum wheat genotypes comes from Mexico (International Maize and Wheat Improvement Center [CIMMYT]), Chile (Chile’s National Agricultural Research Institute [INIA], and Pontifical Catholic University of Chile [PUC]), Spain (Institute of Agrifood Research and Technology [IRTA]), and Tunisia (National Institute of Agronomic Research [INRAT]) breeding programs were used as plant material. The complete list of the germplasm used in this study is available in **Supplementary Table S1**. The field trials were established at Santa Rosa (36° 32′ S, 71° 55′ W; 220 m.a.s.l.) and Los Tilos (33° 70′ S, 70° 70′ W; 530 m.a.s.l.) INIA experimental stations in southern and central Chile, respectively. Considering that durum wheat is grown in Chile from the Valparaíso (central) to Biobío (south) Regions, the sites evaluated in this study represent contrasting production environments. The trials were established during 2021-22 (hereinafter referred to as 22) and 2022-23 (hereinafter referred to as 23) growing seasons under rainfed (r) and irrigated (i) conditions, giving a total of eight environments. The monthly precipitation and temperature data for each site and growing season are shown in **Supplementary Table S2**. For the irrigation treatments, furrow irrigation was used at Santa Rosa (SR) and Los Tilos (LT) locations; one irrigation with 50 mm of water at the end of tillering (Z22) and at the flag leaf stage (Z37) was applied in the two growing seasons in each location. The experimental design was α-lattice with two replications and 15 incomplete blocks per replicate, each containing 15 genotypes. Each entry was planted in plots of five rows of 2 m length and 0.2 m distance between rows. The sowing rate was 22 g m^2,^ and sowing dates were 13 and 18 of August in 2021 and 2022, respectively, at Santa Rosa; 30 of July and 28 of July in 2021 and 2022, respectively, at Los Tilos. The harvest dates were 17 and 19 January 2022 and 2023, respectively, at Santa Rosa, and 10 and 20 January 2022 and 2023, respectively, at Los Tilos.

Plots were fertilized with a mix of 700 kg ha^−1^ with the following element components: 18% P_2_O_5_, 4% N, 15% of K_2_O, 6% of S, 5% of Ca, 0.2% of B and 0.3% of Zn. Fertilizers were incorporated with a cultivator before sowing. At Z20, and Z30, 111 kg ha^−1^ of N was applied. Weeds were controlled with the application of flufenacet + flurtamone + diflufenican (96 g a.i.; Bacara Forte 360 SC, Bayer) as pre-emergence controls and a further application of MCPA (525 g a.i.; Anasac) + metsulfuron-metil (5 g a.i.; Anasac) as post-emergent. To keep the genotypes free of leaf disease as rust (*Puccinia striiformis* and *Puccinia triticina*), two applications were made of the foliar fungicide Miravis Aeon, Syngenta (75 mL ha^−1^; 100.1 g/L azoxystrobin, 125 g/L propiconazole, 75.1 g/L pydiflumetofen). These applications were made before symptoms appeared, to avoid any interference of these diseases in the development of the plants.

#### 2.1.2 Analysis of phenotypic data

Five yield-related traits such as spikes per square meter (SPM), grains per spike (GPS), thousand grain weight (TGW), grain yield (GY), and harvest index (HI) were evaluated. For each yield-related trait, the best linear unbiased predictors (BLUP) were predicted for each site, combined by treatment, and across environments using the META-R software version 6.0 (Alvarado et al., 2020), using the restriction maximum likelihood method (REML). The normal distribution was tested with the Shapiro–Wilk test using the shapiro.test function in R software. Most of the traits in the different data sets followed a normal or approximately normal distribution (**Supplementary Table S3**). The raw data and BLUP values are provided in the supplementary **Supplementary Table S4** and **Supplementary Table S5**, respectively. The linear mixed models used in META-R are implemented in the lme4 package (Bates et al., 2014) and adjusted as follows:


$$Y_{ijk}=u+Rep_{i}+Block_{j}\left(Rep_{i}\right)+Gen_{k}+\epsilon_{ijk}$$

Where $Y_{ijk}$ is the trait of interest, u is the overall mean effect, $Rep_{i}\;$ is the effect of the i th replicate, $Block_{j}\left(Rep_{i}\right)$ is the effect of the j th incomplete block within the i th replicate, $Gen_{k}$ is the effect of the k th genotype and $\;\epsilon_{ijk}\;$ is the effect of the error associated with the i th replication, j th incomplete block, and k th genotype. The distribution was assumed standard with mean zero and variance $\;\sigma_{\epsilon }^{2}$ . All effects, except the overall mean, were treated as random. Regarding the analysis between environments by treatment or between all environments, the terms associated with the environment and the environment x genotype interaction were added to the model (Alvarado et al., 2020). Additionally, phenotypic and genetic correlations among the traits combined across all environments were performed in META-R software version 6.0 (Alvarado et al., 2020).

#### 2.1.3 Analysis of genotypic data

The total genomic DNA of the 225 genotypes was extracted from fresh leaves using Plant/Fungi DNA Isolation Kit (Norgen Biotek, Canada), according to the manufacturer’s instructions. DNA quality and concentration were measured using agarose gel electrophoresis (1%) and Qubit 4.0 (Thermo Fisher Scientific, Inc.). SNP genotyping was performed by Trait Genetics GmbH (http://www.traitgenetics.com/) using the 90K SNP array developed by Wang et al. (2014), of which a total of 51,423 SNPs were distributed on the A and B genome of durum wheat. Monomorphic SNP and SNPs with minor allele frequency (MAF) of less than 5% and those SNPs and accessions containing 10% or more of missing values were removed using TASSEL 5.2 software (Bradbury et al., 2007). Additionally, the genotypes with 10% or more heterozygotes were also eliminated.

#### 2.1.4 Population structure and kinship analysis

SNPs were pruned for linkage disequilibrium (LD) to remove linked loci at the *r* ^2^ > 0.2 using PriorityPruner software (http://prioritypruner.sourceforge.net/) to infer the population structure (Q) and kinship (K) matrices. The number of subgroups among the 225 genotypes was inferred based on a Bayesian model approach implemented in Structure v2.3.4 (Pritchard et al., 2000). An admixture ancestry model with correlated allele frequencies and no prior information of population origin was used. The putative number of subpopulations (*K*) ranged from 1 to 6. Ten replicates were performed for each *K* with a burn-in period of 100,000 steps followed by 1,000,000 MCMC iterations. The optimal *K* value was determined in Structure Harvester (https://taylor0.biology.ucla.edu/structureHarvester/) using the *ad hoc* statistic Δ*K* (Evanno et al., 2005). To confirm the results, a discriminant analysis of the principal components (DAPC) was implemented in ade4 (Chessel et al., 2004) and adegenet (Jombart, 2008) and visualized with ggplot2 (Wickham, 2010) packages in R software. The K matrix was estimated using the ‘Scald_IBS method’ in the TASSEL 5.2 software (Bradbury et al., 2007).

#### 2.1.5 Association analysis

The marker-trait association analysis was carried out in TASSEL 5 (Bradbury et al., 2007) with the following mixed linear model (MLM) proposed by Yu et al. (2006):


y=Sa+Qv+Zu+ϵ

Where y is the vector of BLUPs of each trait at each environment, combined for the two water treatments (irrigated and rainfed), and across all environment, a is the vector of SNP effect (fixed), v is the vector of population structure (Q; fixed), u is the vector of kinship effects (K; random), and ϵ is the vector of residual effects. S , Q , and Z are incidence matrices relating y to a v and u , respectively. A threshold of *p*< 0.001 [−log_10_(*p*) > 3] was used to indicate the significant SNP-trait association (MTA). Manhattan plots were drawn to visualize significant markers using SRplot (Tang et al., 2023), and quantile-quantile (Q-Q) plots to show important p-value distributions. Furthermore, for comparison purposes of the confidence of the MTAs detected using MLM, the general linear model (GLM) and the fixed and random circulating probability unitization model (FarmCPU) were performed on the data sets combined by treatment using the R package rMVP (Yin et al., 2021). MTAs detected for the same trait in at least two environments were reported as stable MTAs and MTAs detected for the same trait in all environments were considered constitutive. Moreover, MTAs were clustered into a unique QTL using the genome LD decay value, where 8.71 Mbp and 7.37 Mbp were used for genome A and B, respectively. The MTAs not in LD were considered as independent QTL.

### 2.2 Physiological study

#### 2.2.1 Yield tolerance index and experimental design

The yield tolerance index (YTI) was calculated for the 225 genotypes established at Santa Rosa during the 2021–22 growing season. This index combines the yield performance of a genotype under drought with its potential yield under irrigated conditions (Ober et al., 2004). The three most tolerant (highest YTI; QUC 3418-2020, QUC 3592-2020, and QUC 3678-2016) and susceptible (lowest YTI; QUC 3319-2020, QUC 3572-2018, and BRESCIA) genotypes were coded from G1 to G6and selected for the evaluation of physiological parameters (**Supplementary Table S6**).

The experiment was performed in a greenhouse under a natural photoperiod with control of temperature and relative humidity at the Pontifical Catholic University of Chile (33°29’ 46.117” S and 70°36’ 27.454” W) using the protocol outlined in Vargas et al. (2016). The experiment consisted of two factors associated with drought treatments: well-watered (WW, 100% of container capacity) and water deficit (WD, 50% of container capacity). The experimental layout was a split plot with five replications, arranging drought treatments as the main factor (2) and the genotypes (6) as the subfactor. The experimental unit was a pot containing 20 seeds arranged randomly for each block. The seeds were sown in rectangular pots (18 x 48 x 14 cm) with a substrate mixture based on mature compost and sand (7:3 v/v). A dose of 10 g of slow-release fertilizer Basacote^®^ plus 6 M (16% N, 8% P_2_O_5_, 12% K_2_O) was applied both at sowing and at the tillering stage, as described by Vargas et al. (2016).

The initial irrigation was the same for all pots. Then, the pots were weighed, and their volumetric water content (VWC) was monitored daily using a Procheck reader with a GS2 METER Group, Inc. USA FDR (Frequency Domain Reflectometry) probe. Watering to replenish the VWC was performed when the volumetric moisture content of the WW 100% treatment decreased to 75% and that of the DW 50% treatment decreased to 15% of the VWC in the pots. The total amount of water used was calculated as the difference between the initial and final weight of the container. The WD treatment began from the full stage of plant tillering (stage 32 of the scale of Zadoks) until the end of the crop.

#### 2.2.2 Gas exchanges and leaf pigments

The net photosynthesis rate (Pn; μmol CO_2_ m^-2^ s^-1^), intracellular CO_2_ content (C_i_; μmol CO_2_ mol air^-1^), transpiration (T; mol H_2_O m^-2^ s^-1^), and stomatal conductance (gs; mol H_2_O m^-2^ s^-1^) were measured using a portable Infrared Gas Analyzer (IRGA) LI-6400/LI-6400XT across treatment between 8:00 to 13:00 on sunny days. All measurements were taken using the fully expanded flag leaf from the appearance of the first awns to the fully emerged spike. The variables CO_2_ (400 ppm), temperature (20°C), and relative humidity (~20%) were monitored. Moreover, the greenhouse radiation at the start of the measurements was close to 200 umol m^-2^ s^-1^, therefore, this radiation was established as a reference. Leaf pigments (chlorophyll content [CC] and nitrogen balance index [NBI]) were non-destructively measured in three flag leaves per pot using a handheld Dualex^®^ Scientific instrument (Force A DX16641, Paris, France). These parameters were measured at three phenological stages: at the end of stem elongation (corresponding to stage 49 of the Zadoks scale, Z49, Zadoks et al., 1974), at the beginning of flowering (stage 61 of the Zadoks scale, Z61), and at the beginning of grain filling (stage 73 of the Zadoks scale, Z73). The leaf water potential (LWP; Scholander et al., 1965) and the relative water content (RWC; Barrs and Weatherley, 1962) were also calculated.

#### 2.2.3 Yield components and data analysis

The different plant tissues were harvested separately per pot and dried at 70°C for two days, and weights were recorded until they remained constant. The following yield components were measured: number of grains, number of empty and productive spikes, number of tillers, number of spikes, 1000-grains weight, pot yield, harvest index, and total dry biomass. A general linear model (ANOVA) analyzed physiological parameters and yield component data. For comparisons of means, the LSD-Fisher test was used with a significant level of 0.05. Additionally, the t-student test was performed to compare treatments. All analyses were performed using the R program (R Core Team, 2023).

### 2.3 Multi-omics approach

Two contrasting genotypes for drought tolerance were identified based on their physiological response and yield components under water stress conditions using data from the 2021–2022 season. Therefore, a new experiment using the drought-tolerant (DT) and drought-susceptible (DS) genotypes was carried out under greenhouse conditions as described above in the “yield tolerance index and experimental design” section to perform the transcriptomic and metabolomic analyses.

#### 2.3.1 RNA extraction and transcriptomic analysis

For the RNA extraction, flag leaf and root tissues were collected at three sampling times: 0, 14, and 30 days after anthesis (61, 75, and 85 on the Zadoks scale, respectively) from the two contrasting genotypes under well-watered (WW) and water-deficient (WD) conditions. Total RNA was extracted from each sample using the NucleoSpin RNA Plant Kit (Macherey-Nagel, Düren, Germany), following the manufacturer’s instructions. RNA quality was evaluated using the Qubit™ RNA IQ Assay Kit with a Qubit™ 4 Fluorometer (Thermo Scientific) and RNA quantity was assessed with a NanoDrop™ Lite spectrophotometer (Thermo Scientific). RNA samples were sequenced using Illumina NovaSeq6000 with an average of 20 million reads per sample (Macrogen, Inc. Seoul). The raw reads were subjected to quality filtering using the FastaQC (v0.11.9) and MultiQC (v1.12) software. Low-quality sequences and adapters were trimmed with the TrimGalore v.0.6.5 (https://github.com/FelixKrueger/TrimGalore), considering those with a Q value > 30 and a sequence size greater than 50 bp. Subsequently, contamination was removed using the Kraken2 software, and a library was built based on high-quality sequences. Filtered reads were aligned against the *Triticum aestivum* reference transcriptome IWGSC RefSeq v1.0 (https://www.wheatgenome.org/) employing the STAR v2.07 software. The differential expression of genes (DEG) was proceeded by DESeq2 (Anders and Huber, 2010). Genes were differentially regulated with a Log2-fold change ≤ - 1 or ≥ 1 and false discovery rate (FDR)< 0.05. The functional annotations of DEGs, the gene sets of Kyoto Encyclopedia of Genes and Genome (KEGG) pathways, and Gene ontology (GO) terms based on biological processes (BP) were employed in gene set enrichment analysis.

#### 2.3.2 Polar metabolite profiling

Leaf root and seed tissues were collected at three sampling times: 0, 14, and 30 days after anthesis (61, 75, and 85 on the Zadoks scale, respectively) in the two contrasting genotypes under WW and WD conditions. The extraction and derivatization of polar metabolites were determined according to the protocol of Hatoum et al. (2014), slightly modified by Fuentealba et al. (2017). Briefly, 100 mg of frozen tissue powder was mixed with 500 μL of cold methanol, and 20 μL of 2910 ng L^−1^ phenyl β-D-glucopyranoside, and the sample was incubated at 70°C for 15 min with shaking (Labnet International Inc., Edison, NJ, USA). After centrifugation at 17–000 g for 20 min, 100 μL of supernatant was dried under a stream of nitrogen gas. The derivatization consisted of methoximation and trimethylsilylation reactions. GC-MS methods were performed, one for more concentrated compounds, such as sugars, and the other for less concentrated compounds, such as organic and amino acids. For both methods, the injector and interface temperatures were 220°C and 280°C, respectively, and 1 μL of sample was injected. Helium was used as carrier gas with a constant flow of 1 mL min^-1^. Mass spectra in the 50–600 m/z range were recorded at a scanning speed of 2.66 scan cycles per second. The MS ion source and quadrupole temperatures were 230°C and 150°C, respectively. The method for more abundant compounds included an injection with a split ratio of 1:150, and the oven temperature was programmed to start at 120°C (for 1 min), increased to 300°C at 10°C min^−1,^ and then held for 6 min. The method for less abundant compounds used a splitless injection mode, and the oven temperature was set to start at 50°C (for 1 min), increased to 310°C at 10°C min^−1,^ and then held for 13 min. The chromatographic peaks were deconvolved and identified by comparing retention times and mass spectra to a home-built library of commercial standards and the NIST14 library using Mass Hunter Quantitative software (Agilent Technologies, Santa Clara, CA, USA). To obtain a relative response of each compound, the peak area data were corrected using the peak area of phenyl β-D-glucopyranoside (as internal standard), the sample dry weight, and a quality control (QC) sample representative of all samples.

Partial Least Squares Discriminant Analysis (PLS-DA) was performed on the normalized dataset using the MetaboAnalyst 5.0 software (https://www.metaboanalyst.ca/MetaboAnalyst/). PLS-DA analysis was used with metabolites as predictor variables and the drought and genotype treatments as response variables. Variables were mean-centered and weighted by the standard deviation to assign an equal variance. Variable Important in Projection (VIP) scores were used to filter PLS-DA results to determine essential features.

#### 2.3.3 Transcriptomics and metabolomics integration

Normalized expression/abundance values of transcripts and metabolites were scaled considering the mean-centered and divided by the standard deviation of each variable, which was used to perform multi-omics integration analysis. The R package mixOmics v6.20.0 (Rohart et al., 2017) was used to perform the multiblock PLS-DA, considering the average representation space between all datasets with the *block.plsda* function described for the DIABLO software (Singh A. et al., 2019). Correlations between omics datasets (transcriptomics and metabolomics) were calculated and plotted using the *plotDiablo* function of the same package. Finally, the *tune.block.splsda* function was used to identify the minimum number of variables that explain the sample dispersion of each variate, and correlations between selected variables were plotted using the *circosPlot* function considering a correlation cutoff = 0.8.

## 3 Results

### 3.1 Phenotypic variation for yield-related traits

A panel of 225 durum wheat genotypes were grown under rainfed and irrigated conditions in two locations (Los Tilos and Santa Rosa, central and south of Chile, respectively) for two consecutive years (the 2022 and 2023 seasons), and five yield-related traits were measured, such as spikes per square meter (SPM), grains per spike (GPS), thousand grain weight (TGW), grain yield (GY), and harvest index (HI). The combined analysis of variance (ANOVA) across all sites showed significant effects (*p*< 0.05) of genotypes (G), environments (E), and their interactions (G×E) for all traits, except for grains per spike (GPS) and spike per square meter (SPM), in which the G×E factor was not significant (**Supplementary Table S7**). The E effect explained most of the variation for grain yield (GY; 79.8%) and 1000-grains weight (TGW; 63.2%), harvest index (HI; 55.8%), and GPS (54.7%), whereas the G×E effect explained the most significant variation for SPM (45.5%). Moreover, the G×E interaction contributed more to the total variability than the G effect for all traits except for TGW, which is in accordance with the value of its broad-sense heritability value of *H^2^ =* 0.882. Under rainfed conditions, the E effect explained over 52% of the variation for all yield-related traits, indicating that the environment has a key impact on durum wheat yield. Phenotypic variation was found for all traits across the 8 environments (**Supplementary Figure S2**). The highest average GY was recorded in the SR_2022 site (9.46 t ha^-1^) under irrigated conditions, whereas SR_2023 had the highest average GY (4.3 t ha^-1^) under rainfed conditions. On average, a 36% reduction in yield was observed at the two locations during the two growing seasons due to drought (**Supplementary Table S8**). The grand mean, variance components, and heritability for each trait are shown in **Table 1**. The phenotypic variances were higher than the genotypic variances in all the traits in the combined analysis across all environments. However, the genotypic variances were higher than their corresponding environmental variances for GPS (7.95) and TGW (10.02), indicating that these traits have greater genetic variability within the traits evaluated in the durum wheat panel. In addition, the GPS and TGW were the ones that presented the highest heritability with values of *H^2^ =* 0.825 and *H^2^ =* 0.882, respectively. These results show that GPS and TGW have greater potential for genetic gains due to selection in breeding programs using this durum wheat panel. On the other hand, the harvest index (HI) was the trait that presented the lowest genetic variation (0.000) and heritability (*H^2^* = 0.416), being a trait with no potential to be used as a selection criterion to obtain genetic gains.

**Table 1:** Grand mean, variance components, and heritability for grain yield (GY), spikes per square meter (SPM), harvest index (HI), grains per spike (GPS), and thousand grain weight (TGW) under irrigated, rainfed, and across all environments.

Environment	Statistic	GY	SPM	HI	GPS	TGW
Irrigated	*H* ^2^	0.509	0.288	0.083	0.700	0.804
	$\sigma_{g}^{2}$	35.026	569.738	0.000	8.910	10.951
	$\sigma_{ge}^{2}$	39.965	961.071	0.000	1.656	1.837
	$\sigma_{e}^{2}$	190.225	6548.690	0.005	27.188	17.625
	Grand Mean	75.969	423.052	0.308	28.696	48.192
Rainfed	*H* ^2^	0.269	0.395	0.438	0.665	0.774
	$\sigma_{g}^{2}$	4.758	542.927	0.001	6.106	9.173
	$\sigma_{ge}^{2}$	11.624	159.782	0.001	1.553	3.117
	$\sigma_{e}^{2}$	80.104	4676.959	0.003	21.470	15.143
	Grand Mean	48.947	362.107	0.249	26.238	39.094
Across	*H* ^2^	0.600	0.481	0.416	0.826	0.882
	$\sigma_{g}^{2}$	17.856	524.080	0.000	7.953	10.022
	$\sigma_{ge}^{2}$	27.582	592.283	0.000	1.252	2.528
	$\sigma_{e}^{2}$	135.166	5613.349	0.004	24.303	16.334
	Grand Mean	62.458	392.580	0.282	27.467	43.643

*H* ^2^: broad-sense heritability; $\sigma_{g}^{2}$ : genotype variance; $\sigma_{ge}^{2}$ : genotype × environment variance; $\sigma_{ge}^{2}$ : residual variance.

The genetic and phenotypic relationship among traits under irrigated, rainfed, and across environments showed significant (*p*< 0.05) correlations among most of the traits (**Supplementary Table S9**). Under well-water (WW) conditions, the strongest genetic positive associations were obtained between GPS and HI (*r* = 0.999; *p*< 0.05) and GPS and GY (*r* = 0.611; *p*< 0.05), while the strongest negative correlation was found between TGW and HI (*r* = -0.771; *p*< 0.05). The highest positive phenotypic correlation was found between GPS and GY (*r* = 0.515; *p*< 0.05), while the negative one was between TGW and GPS (*r* = -0.542; *p*< 0.05). Under water deficit (WD) conditions, the highest positive genetic correlation was between HI and GY (*r* = 0.924; *p*< 0.05), while the highest negative correlation was between TGW and GPS (*r* = -0.771; *p*< 0.05). The highest positive and negative phenotypic correlations were found between HI and GY (*r* = 0.445; *p*< 0.05) and TGW and GPS (*r* = -0.553; *p*< 0.05), respectively. Overall, GPS recorded one of the highest and most consistent genetic and phenotypic correlations with GY in both water regimes and across all environments.

### 3.2 Markers distribution and population structure

12,498 SNP markers distributed across the 14 chromosomes, primarily positioned in the regions close to the telomeres, were retained after filtering criteria (**Supplementary Figure S3**). Out of the total markers, 5,969 and 6,529 SNPs were located on the genomes A and B, respectively. On genome A, the number of markers varied from 631 to 1,061 on chromosomes 4A and 7A, respectively, with an average of 852 SNPs per chromosome. In the B genome, the number of markers ranged from 644 to 1,134 on chromosomes 4B and 2B, respectively, with an average of 932 SNPs per chromosome. The physical chromosome length ranged from 593.5 to 829.5 Mpb on chromosomes 1A and 3B, respectively. The highest density of markers was found on chromosome 1B with ~1.57 SNP/Mpb (**Supplementary Table S10**).

Based on the structure and DAPC analyses, using 5,258 SNPs retained after filtering by LD (*r* ^2<^ 0.2), the 225 durum wheat lines were divided into two groups. Population structure analysis indicated *K* = 2 as the most probable number of subpopulations. Based on the membership coefficient (Qi > 0.8), 18 lines (7 from INIA [Chile’s National Agricultural Research Institute] and 11 from CIMMYT [International Maize and Wheat Improvement Center]) were grouped into group 1, while 194 lines (87 from INIA, 98 from CIMMYT, 3 from PUC, 2 from INRAT, and 4 IRTA) were classified into group 2. This result indicates that the durum wheat lines were not grouped according to their genebank origin. Conversely, five sub-populations were inferred according to DAPC analysis by the origin of the durum wheat lines (**Supplementary Figure S4**). The first and second principal components (PC) explained 58.3% and 20.5% of the total variation, respectively, in which the PC1 allows the differentiation of lines from the INIA and CIMMYT gene banks. Finally, the results from both Structure and DAPC analyses showed an admixture between the genotypes of the different germplasm banks, mainly those of INIA and CIMMYT, common in durum wheat lines developed in breeding programs. The subsequent association analysis was performed using the two principal components.

### 3.3 Linkage disequilibrium and genome-wide association

The LD decay was similar in both genomes. In genome A, the LD decayed at values less than *r* ^2^< 0.2 at an approximate distance of 8.71 Mpb; in genome B, the LD decayed decay at 7.37 Mbp (**Supplementary Figure S5**).

**Figure 1:**
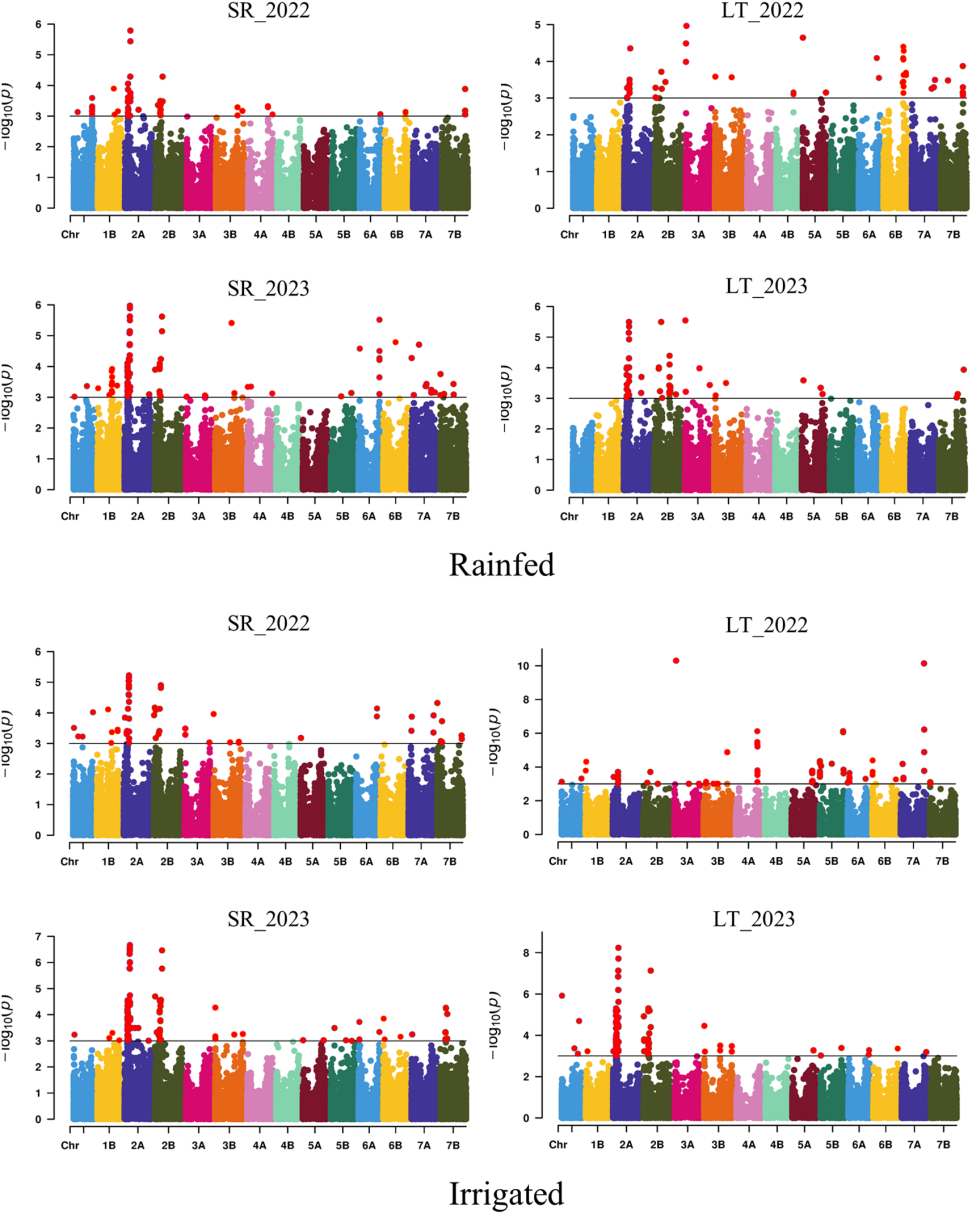
Manhattan plot showing the SNPs significantly associated with the yield-related traits evaluated across eight environments. SR, Santa Rosa; LT, Los Tilos; 2022, 2021/22 growing season; 2023, 2022/23 growing season; Rainfed and Irrigated with 50 mm at the end of tillering and the flag leaf stage.

Based on the individual analysis by environment, a total of 772 significant SNPs (-log_10_P > 3) distributed across the 14 chromosomes of the durum wheat genome were associated with the five yield-related traits through the eight environments using the MLM model (**Supplementary Table S11**). Marker-trait associations (MTAs) were 331 and 441 under rainfed and irrigated conditions, respectively. The chromosomes with the highest number of MTAs were 2A (351) and 2B (102), whereas the lowest number of MTAs were 4B (2), 4A (18), and 5A (18) (**Figure 1**). In this context, 300, 206, 115, 88, and 63 MTAs were identified for TGW, GPS, HI, SPM, and GY, respectively. The phenotypic variation explained (PVE) for each MTA ranged from 5% (BS00000297_51 on chromosome 2A) to 24.3% (Excalibur_c12446_155 on chromosome 3A), both for HI. A total of 57 and 33 MTAs were stable in more than one environment under irrigated and/or rainfed conditions, respectively. Within the stable MTAs, eight were detected exclusively under rainfed conditions, which were clustered into two genomic regions associated with TGW on chromosomes 2A (*QTN_2A.TGW.1_R*) and 3A (*QTN_3A_TGW.1_R*), explaining between 5.11% and 11.07% of the phenotypic variance. Under irrigated conditions, 32 stable MTAs were detected, clustered into twelve genomic regions on chromosomes 1A, 2A, 2B, and 5A. All genomic regions were associated with TGW and GPS, explaining between 5.15% and 10.41% of the phenotypic variation, except a quantitative trait loci (QTL) (*QTN_2B_GY.1_I*) located on chromosome 2B at 56.78 Mpb that explained between a 5.80% and 7.42% of the GY variation (**Supplementary Table S12**). Finally, nine constitutive MTAs were identified across all environments (**Table 2**), which were grouped into three QTL associated with TGW and GPS located on chromosomes 2A (*QTN_2A_TGW/GPS.1*; *QTN_2A_TGW/GPS.2*) and 2B (*QTN_2B_TGW/GPS.1*), explaining between 5.15% and 14.29% of the variation.

**Table 2:** Nine constitutive MTAs were identified across all eight environments, grouped into three drought-tolerant QTLs.

Marker	Trait	Chr	Pos (Mpb)	PVE (%)	Name QTL
GENE-1342_238	TGW;GPS	2A	146.959587	5.15 - 13.6	QTN_2A_TGW/GPS.1
RAC875_rep_c74200_1415	TGW;GPS	2A	146.962374	5.15 - 13.6	
wsnp_CAP11_c1787_968410	TGW;GPS	2A	146.962374	5.15 - 13.6	
GENE-1319_146	TGW;GPS	2A	151.25509	5.62 - 14.29	
TA001351-1193	TGW;GPS	2A	151.258908	5.62 - 14.29	
BobWhite_c2532_966	TGW;GPS	2A	154.415628	5.62 - 14.29	
Tdurum_contig27887_55	TGW;GPS	2A	154.415628	5.62 - 14.29	
wsnp_BG607088A_Ta_1_1	TGW;GPS	2A	156.762886	5.39 - 13.21	QTN_2A_TGW/GPS.2
GENE-1181_694	TGW;GPS	2B	209.088615	5.62 - 14.29	QTN_2B_TGW/GPS.1

Chr, Chromosome; Pos, position; PVE, percentage of the phenotypic variation explained.

Based on the combined analysis by water treatment, the GLM model, as measured by the Q-Q plot, did not show a pronounced deviation from the expected distribution of P values in the tail area, indicating poor control of false positives and negatives. In contrast, while the FarmCPU model better controlled false positives and negatives, the MLM model used in this study also provided adequate control (**Supplementary Figure S6**). Therefore, our MTAs detected using MLM are reliable. A total of 152 significant MTAs (-log10P > 3) were found for the five yield-related traits under irrigated conditions, with two for GY, 13 for SPM, 24 for HI, 54 for GSP, and 55 for TGW (**Supplementary Table S13**). The percentage of phenotypic variation explained by each MTA varied from 5.23% (Kukri_rep_c108875_85 marker on chromosome 2B) to 16.49% (Tdurum_contig27887_55 marker on chromosome 2A), both markers associated with TGW and GPS. Under rainfed conditions (**Supplementary Table S14**), a total of 107 MTAs were detected for GY (10), SPM (2), HI (7), GPS (48), and TGW (40). The marker BS00066873_51 with HI located on chromosome 2B explained the lowest percentage of phenotypic variance, while the Tdurum_contig27887_55 (2B) marker associated with TGW and GPS explained the highest percentage (15.69%). Finally, under the combined analysis of both conditions (**Supplementary Table S15**), a total of 133 MTAs were detected for GY (2), SPM (2), HI (5), GPS (64), and TGW (60). The percentage of phenotypic variation explained by each MTA varied from 5.13% (Excalibur_c4847_631 marker on chromosome 2A) to 18.03% (Tdurum_contig27887_55 marker on chromosome 2A), both markers associated with TGW and GPS. Interestingly, the Tdurum_contig27887_55 marker located at 156,366,746 bp on chromosome 2B, which was associated with GPS and TGW, it was consistently the one that explained the greatest phenotypic variation under irrigated, rainfed, and in the combined analysis of both conditions.

### 3.4 Physiological effects of drought

The summary of the physiological parameters evaluated for the six genotypes can be visualized in **Table 3**. According to the results, the G3 (BRESCIA) and G4 (QUC 3678-2016) genotypes were the most and least affected, respectively, due to water deficit (WD) treatment. Therefore, these genotypes are appropriate candidates for studying mechanisms associated with susceptibility (BRESCIA) and tolerance (QUC 3678-2016) in the durum wheat panel.

**Table 3:** Effects of water treatment on different physiological parameters evaluated in six contrasting drought tolerant/susceptible durum wheat genotypes.

Category	Parameter	Measure	G1	G2	G3	G4	G5	G6
Gas exchange	Pn	Fully-expanded leaf			*			
	T	Fully-expanded leaf			*	*		*
	Gs	Fully-expanded leaf			*	*		*
	C_i_	Fully-expanded leaf			*			*
Leaf pigment	CC	End of stem elongation			*		*	
		Beginning of flowering	*					*
		Beginning of grain filling	*	*	*			
	NBI	End of stem elongation			*			
		Beginning of flowering	*					*
		Beginning of grain filling		*	*			
Water status	LWP	End of stem elongation			*			
		Beginning of grain filling	*		*			
	RWC	End of stem elongation						
		Beginning of grain filling						
Yield	N° grains	Harvest	*	*	*	*		
	TGW	Harvest	*		*			
	Yield	Harvest		*	*			
	Number of tillers	Harvest		*	*	*	*	*

*Significant differences between treatments according to the Tukey test (p<0.05).

Regarding the gas exchange parameters studied, the genotype and the genotype x water treatment interaction were significant only for stomatal conductance (gs) according to the ANOVA (**Supplementary Table S16**), in which the genotypes that showed the highest gs under water-deficit (WD) conditions were G1 (0.26 mol H_2_O m^-2^ s^-1^) and G2 (0.24 mol H_2_O m^-2^ s^-1^), while the lowest values of gs were found in G3 (0.03 mol H_2_O m^-2^ s^-1^) and G5 (0.07 mol H_2_O m^-2^ s^-1^). The genotypes that most and least reduced gs due to WD treatment were G3 (75%) and G6 (7.14%), respectively. The G4 genotype had a gs reduction of 66.66%, and, surprisingly, the G1 genotype increased gs by 136.36%. The highest net photosynthetic rate (Pn) was observed in G4 (Pn = 6.3 μmol CO_2_ m^-2^ s^-1^) and G2 (Pn = 8.73 μmol CO_2_ m^-2^ s^-1^) for well-watered (WW) and WD conditions, respectively. The G3 and G4 genotypes reduced Pn due to WD treatment by 32.14% and 37.14%, respectively. According to the t-student test performed, significant differences (p< 0.05) were found between treatments for C_i_ and T in G3 and G4 genotypes. The G3 genotype was the most affected with respect to the intracellular CO_2_ concentration (C_i_) and transpiration (T) with a reduction of 23.8% and 69%, respectively. While the G4 genotype had a reduction of 14.29% and 60.94% for C_i_ and T, respectively.

At the end of the stem elongation stage (Z 49), nitrogen balance index (NBI) did not show significant differences between genotypes and water treatments according to the ANOVA (**Supplementary Table S17**). The G3 genotype was the most affected with a reduction of 25.38%, while the G4 genotype had a 4% reduction. Chlorophyll content (CC) was significantly reduced due to WD treatment, with genotype G6 standing out with a 26% reduction, followed by G3 with 16%. The G4 genotype was little affected due to WD treatment and had a reduction of 6.32% in CC. During flowering (Z 61, Zadoks et al., 1974), no differences were found between genotypes and between water treatments. However, NBI and CC increased due to WD treatment in the G3 and G4 genotypes. The G3 had an increase of 3% and 5% for NBI and CC, respectively. While the G4 genotype increased by 4.5% and 6% for NBI and CC, respectively. At the beginning of grain filling (Z 73), only 4 genotypes (G1, G2, G3, and G4) were evaluated due to technical limitations. Significant differences were found for NBI and CC only for the factor treatment. The G3 genotype experienced a more significant reduction for NBI (39.76%) and CC (42.69%) due to WD treatment. It is worth noting that G4 had the lowest reduction between treatments associated with NBI (11%) and CC (18%).

At the end of stem elongation (Z 49) and during beginning of flowering or grain filling (Z 60), no significant differences were observed between genotypes for leaf water potential (LWP). Compared to the control, the G3 genotype significantly reduced its LWP by 57.62% and 53.96% at stages Z 49 and Z 60, respectively. The G4 genotype had a 1.5% reduction at stage Z 49 and an 11.59% increase at stage Z60 for LWP (**Supplementary Table S18**). For relative water content (RWC), no significant differences were identified between genotypes and treatments at stages Z 49 and Z 60. However, the genotype x treatment interaction was substantial at stage Z 49. The G3 genotype had a reduction of 1% and 19% at Z 49 and Z 60 stages, respectively. While the G4 genotype had a reduction of 1% in both stages of development. Finally, for the yield-related traits, there were significant differences between genotypes and between treatments, in which the G1, G5, and G3 genotypes were the most affected under WD conditions. In contrast, the G6 and G4 genotypes were the least affected. The grain yield was reduced by 67.5% and 38.09% for G3 and G4 genotypes, respectively (**Supplementary Table S19**).

### 3.5 Overall gene expression profile under drought stress

Gene transcription from leaf and root tissues at 0-, 14-, and 30- days post anthesis (DPA) under WW and WD conditions were compared to investigate the molecular responses of the two contrasting genotypes of durum wheat to drought previously reported. An average of 27,278,116 reads were generated, of which 26,294,562 were retained after the quality and trimming filters, meaning 96.3% sequencing integrity. In total, 85% of sequences were aligned to the genome of *Triticum turgidum* Svevo v1.0, within which 81.43% had an alignment to a single locus while 4% showed an alignment to multiple loci. Overall, there was a higher amount of differentially expressed genes (DEGs) in the root compared to the leaf for comparisons between genotypes and treatments at the three developmental stages studied. 11,176 DEGs were identified in the leaf, of which 4,819 were up- and 6,357 down-regulated. In root, 12,229 DEGs were identified, of which 5,489 were up- and 6,740 down-regulated. In the leaf, a more significant number of DEGs were determined by the comparison between genotypes (9,173) than between treatments (2,003). In root, the number of DEGs identified by comparing genotypes and treatments was 6,949 and 5,280, respectively. It was confirmed through principal component analysis (PCA) that the transcriptome data of the leaf could separate the G3 (drought-susceptible, DS) and G4 (drought-tolerant, DT) genotypes by PCA1 (39% of variance) and the drought treatment by PC2 accounting for 22% of variance. At root, there was no clear separation between genotypes and treatments, where PC1 and PC2 explained 36% and 15% of the variance, respectively (**Supplementary Figure S7**).

**Figure 2:**
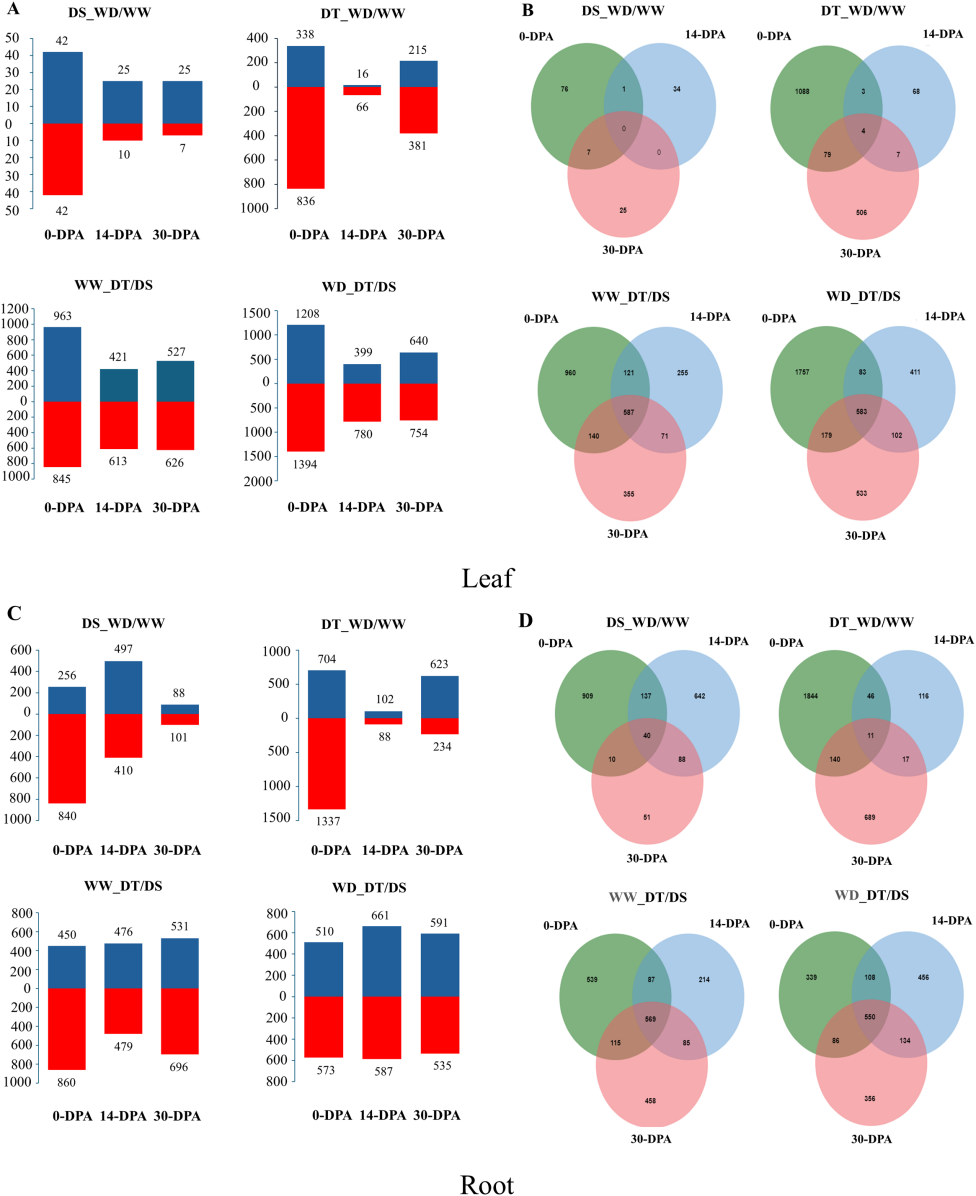
A total number of up-and down-regulated genes identified by the comparison of well-water (WW) and water deficit (WD) conditions in the drought-tolerant (DT) and drought-susceptible (DS) genotypes and the comparison DT/DS genotypes under WW/WD conditions at 0-, 14-, and 30-days post anthesis (DPA) in leaf **(A, B)** and root **(C, D)**.

The summary of the total number of up-and down-regulated genes identified by the comparison of WD/WW in the two contrasting genotypes and DT/DS under different water conditions at the three developmental stages in the leaf and roots is shown in **Figure 2**. A large proportion of drought-responsive genes were induced in leaf at 0-DPA in both genotypes after drought treatment, and the number of drought-responsive genes in the DT genotype (1,174 DEGs) was much higher than in the DS genotype (84 DEGs) after drought stress exposure. 151 and 1852 DEGs were identified by comparing WD/WW, with 92 and 569 up-regulated genes and 59 and 1283 down-regulated genes for DS and DT genotypes, respectively (**Figure 2A**). Moreover, four DEGs were constitutive across 0 to 30-DPA in the DT genotype (**Figure 2B**), which were TRITD2Bv1G146530 (encodes a plastid-localized arogenate dehydratase involved in phenylalanine biosynthesis), TRITD4Bv1G179760 (encodes a chloroplast RNA-binding protein), TRITD5Av1G000200 (encodes a grain softness protein), and TRITD5Bv1G186950 (Serine protease inhibitor (SERPIN) family protein). Interestingly, the TRITD5Bv1G186950 and TRITD5Av1G000200 genes were down-regulated (Log_2_FC< -20) at 0-DPA, and then at 14- and 30-DPA were highly up-regulated (Log_2_FC > 20). Additionally, 583 DEGs were compared to the two genotypes under WD conditions.

Different expression patterns across the three developmental stages studied were observed in the root. In the DS genotype, a more significant amount of DEGs were observed at 0-DPA (1,096) and 14-DPA (907), whereas a greater amount of DEGs were observed at 0-DPA (2,041) and 30-DPA (857) in the DT genotype, which were much higher compared to the DS genotype. 2192 and 3088 DEGs were identified by comparing WD/WW, with 841 and 1429 up-regulated genes, 1351 and 1659 down-regulated genes for DS and DT genotypes, respectively (**Figure 2C**). 11 DEGs were constitutive in the DT genotypes (**Figure 2D**). The total number of DEGs under WD was higher than that under WW conditions. Finally, 569 and 550 DEGs were constitutive in the three developmental stages under WW and WD conditions, respectively.

### 3.6 Gene classification by gene ontology

Since the reference genome of *T. turgidum* does not have a robust functional annotation, the functional classification was performed from an alignment against the genome of *A. thaliana*. Gene ontology (GO) analysis was based on classifying DEGs into various biological processes by overrepresenting these genes within the genome. GO analysis showed genotype-, time-, and tissue-specific results, revealing that some important biological processes occur differently in the two contrasting genotypes evaluated under water stress. However, some biological processes were common, highlighting cell death, response to salicylic acid, and biosynthesis of secondary metabolites.

**Figure 3:**
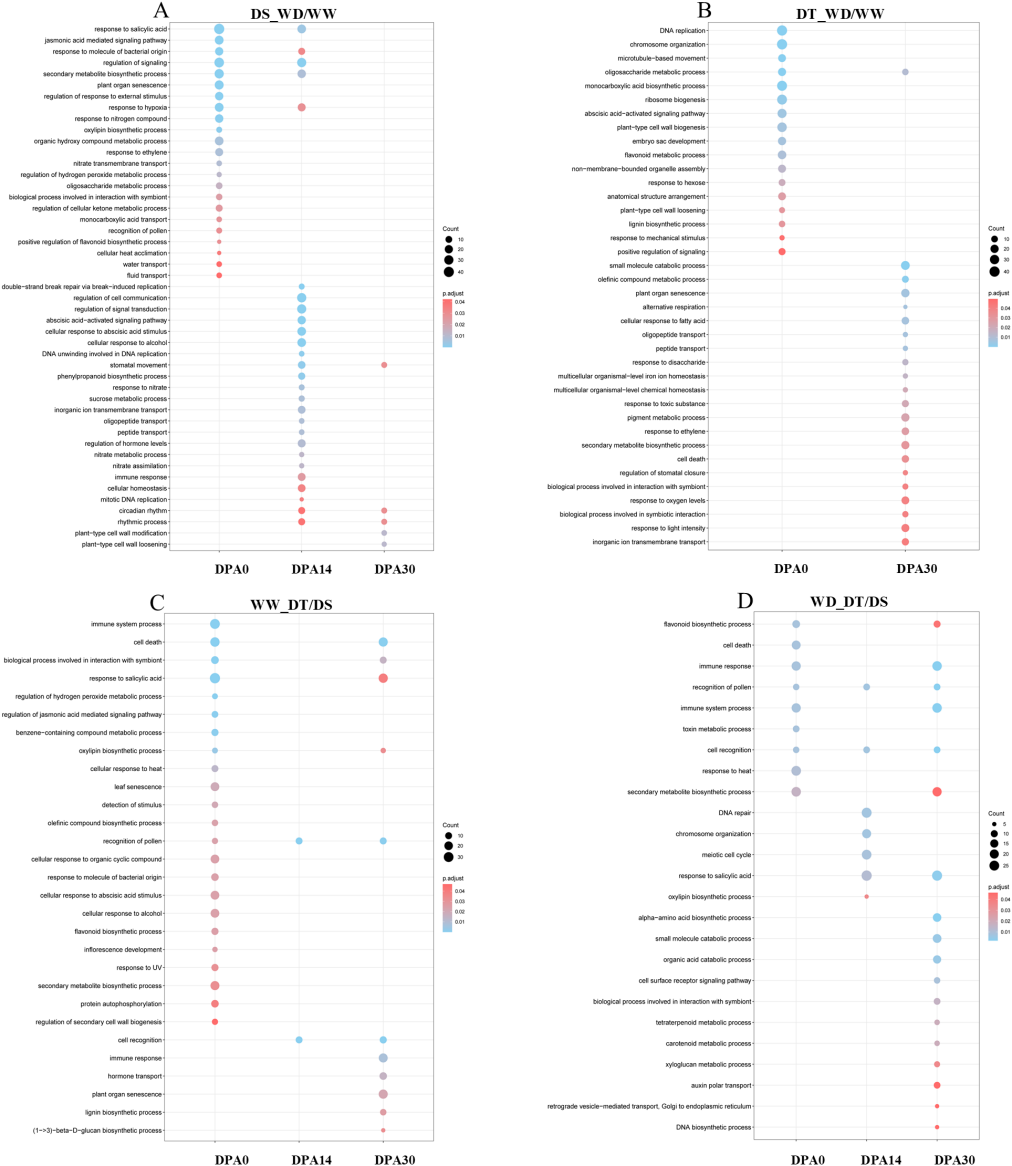
Gene ontology (GO) enrichment analysis in the root. Comparison between well-water (WW) and water deficit (WD) treatments for drought-susceptible (DS; **A**) ​​and drought-tolerant (DT; **B**) genotypes. Comparison between DS and DT genotypes under WW **(C)** and WD **(D)** conditions.

In leaf tissue, the DS genotype only showed enrichment at 14 DPA, whereas the DT genotype exhibited enrichment at 0 DPA and 30 DPA (**Supplementary Figure S8**). Additionally, genes associated with the DS genotype were classified into fewer biological processes than the DT genotype, with most genes related to gibberellin response and ribosome biogenesis. In contrast, the functional annotation of genes associated with the DT genotype involved a wide range of biological processes, highlighting cell death, salicylic acid response, immune response, and secondary metabolite biosynthesis. Comparing the two genotypes under WW conditions, the overrepresentation of genes associated with senescence, monocarboxylic acid biosynthesis, aromatic compound catabolism, and cell death was found. Under WD conditions (WD_DT/DS), the enriched processes were senescence, hypoxia, cell death, and salicylic acid response at 0-DPA. In later developmental stages, the persistence of genes associated with senescence and citric acid response was observed, as well as the appearance of genes related to secondary metabolite biosynthesis and metabolic processes associated with hydroxyl group-containing compounds. The DS genotype showed gene enrichment across all three times studied in root tissue. Many genes associated with various biological processes were identified, including salicylic acid response, signaling regulation, secondary metabolite biosynthesis, senescence, response to external stimuli, ABA signaling pathway, and cellular communication regulation (**Figure 3A**). In the DT genotype, gene enrichment was observed in processes unrelated to drought stress response. In early developmental stages, genes were primarily associated with DNA replication, chromosomal organization, microtubule movement, ribosome biogenesis, cell wall biogenesis, monocarboxylic acid biosynthesis, and ABA signaling pathway. In contrast, in later developmental stages, genes were mainly classified into catabolic processes and senescence (**Figure 3B**). In both genotypes under WW conditions, gene enrichment was observed in flavonoid biosynthesis, secondary metabolite biosynthesis, and processes related to the plant immune system (**Figure 3C**). Under WD conditions, gene overrepresentation was associated with immune response, cell death, and salicylic acid response (**Figure 3D**).

### 3.7 Metabolite profiling under drought stress

**Figure 4:**
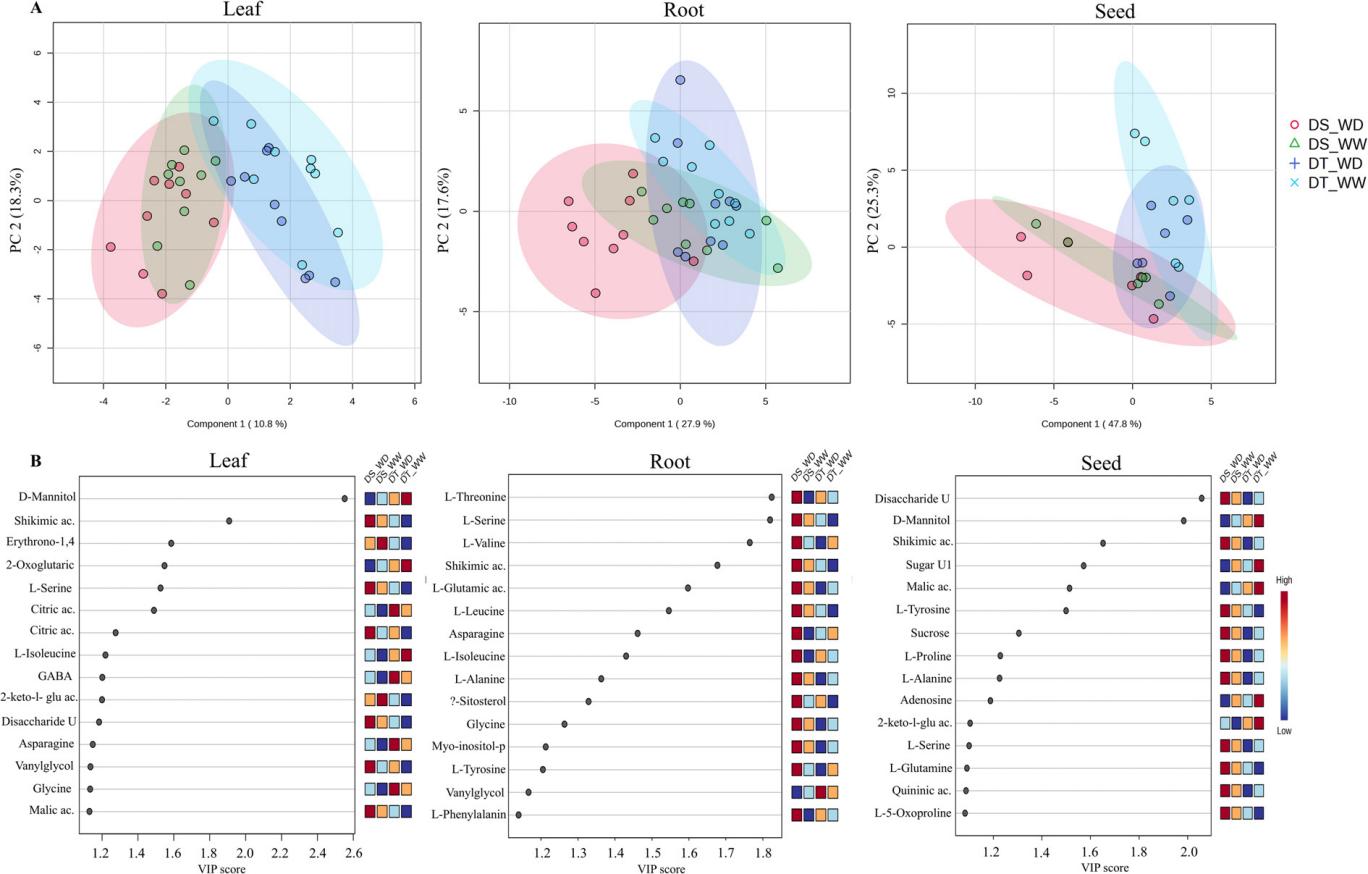
Partial least squares-discriminant analyses (PLS-DA) with the two first components **(A)** and variable’s importance (VIP scores) of secondary metabolite profiles **(B)** of leaf, root, and seed of the drought-tolerant (DT) and drought-susceptible (DS), genotypes under well-water (WW) and water deficit (WD) conditions at 0-, 14- and 30-DPA.

To investigate the metabolic response under drought in selected genotypes (G3 and G4), the relative abundance of polar metabolites in leaf, root, and seed tissues was determined at 0-, 14-, and 30-DPA by gas chromatography/mass spectrometry (GC–MS) (**Figure 4**). Overall, 43 metabolites were identified and quantified, 37 were identified as known compounds, and 6 metabolites were unknown. Of these metabolites, most were amino acids, sugars, and organic acids. Moreover, the highest number of known compounds were present in leaf (42), then in root (41) and seeds (38). A Partial Least Squares-discriminant Analysis (PLS-DA) showed that the metabolite profiles between DT and DS genotypes differed (**Figure 4A**), which suggests a distinction in the metabolite accumulation response due to genotypic differences. The score plots explained 29.1% (leaf), 45.5% (root), and 73.1% (seed) of the variability with the two first components. Variable importance of projection (VIP) scores were determined for displaying the 15 most important metabolites involved in the discrimination between genotypes and treatment (**Figure 4B**). Among the five most contrasting metabolites in the leaf are D-Mannitol, shikimic acid, erythrono-1,4-lactone, (Z), 2-oxoglutaric acid, and L-Serine. The root had mainly amino acids such as L-threonine, L-Serine, L-valine, L-glutamic acid, and shikimic acid. In contrast, a disaccharide unknown, D-Mannitol, shikimic acid, unknown sugar, and malic acid were the five most contrasting metabolites in seeds. Only four metabolites showed higher abundance in the DT genotype under WD conditions in the leaf (citric acid, 2-Keto-l-gluconic acid, glycol, and malic acid). Interestingly, vanylglycol was the only metabolite with a high abundance in roots, while no metabolite had the highest abundance in the DT genotype under WD conditions in seeds.

Further analysis of individual metabolites based on fold-change (FC) of metabolite abundance between the comparison of WD/WW in the two genotypes and DT/DS under the two water conditions at different developmental stages in leaf and roots is shown in **Supplementary Figures S9**, **S10**. Across the three time points in the leaf, the total number of differential abundance of metabolites (DAMs) was 30 and 35 in the DT and DS genotypes, respectively. Only two (asparagine and quininic acid) and one (L-Proline) metabolites were identified at all three times (constitutive) in DT and DS genotypes due to drought treatment, respectively. Further, 33 and 43 DAMs were determined by comparing DT/DS genotypes, with 24 and 25 up-concentrated and 9 and 18 down-concentrated metabolites for WW and WD conditions, respectively. Under WD conditions, a similar number of DAMs was observed at 0-DPA (13) and 14-DPA (12), while the number was higher (18) in the later stage (30-DPA). Moreover, five (D-mannitol, asparagine, aspartic acid, L-glutamic acid, malic acid) metabolites were constitutive across 0 to 30-DPA in the comparison between the two genotypes under WD conditions, and only citric acid was under WW conditions.

A higher number of DAMs was determined in the root than in the leaf. In this sense, 41 and 49 DAMs were determined across the three phenological stages in the DT and DS genotypes under WD conditions, respectively. Furthermore, more constitutive DAMs were identified in roots compared to leaves. Nine metabolites were constitutive in the DS genotype, which were amino acids (L-Proline, Asparagine, L-Glutamine, L-Threonine, L-Valine, and L-Phenylalanine), sugars (D-Fructose, and D-Glucose) and the polyol D-Mannitol. In the DT genotype, the metabolites constitutive were organic acids (Quininic acid, Aconitic acid, 2-Oxoglutaric acid, and Quininic acid), the amino acid L-Proline, and an unknown disaccharide. Comparing both contrasting genotypes, a similar number of metabolites were up-concentrated under WW (14) and WD (15) conditions.

Interestingly, more metabolites were down-concentrated under WD (41) than WW (8) conditions. Four metabolites were constitutive under WD conditions: organic acids (aconitic, aspartic, and glyceric acid) and L-valine. In contrast, the D-Mannitol and 5-O-Feruloylquinic acid were constitutive under WW conditions. These results indicate that the metabolic response of durum wheat under water stress conditions is modulated at a specific time, indicating the differential abundance of metabolites at different developmental stages. However, constitutive metabolites are up-and down-concentrated at different times (0- to 30-DPA), which can be used as biomarkers associated with drought stress in leaves and roots.

### 3.8 Integration of transcriptomic and metabolomic data

**Figure 5:**
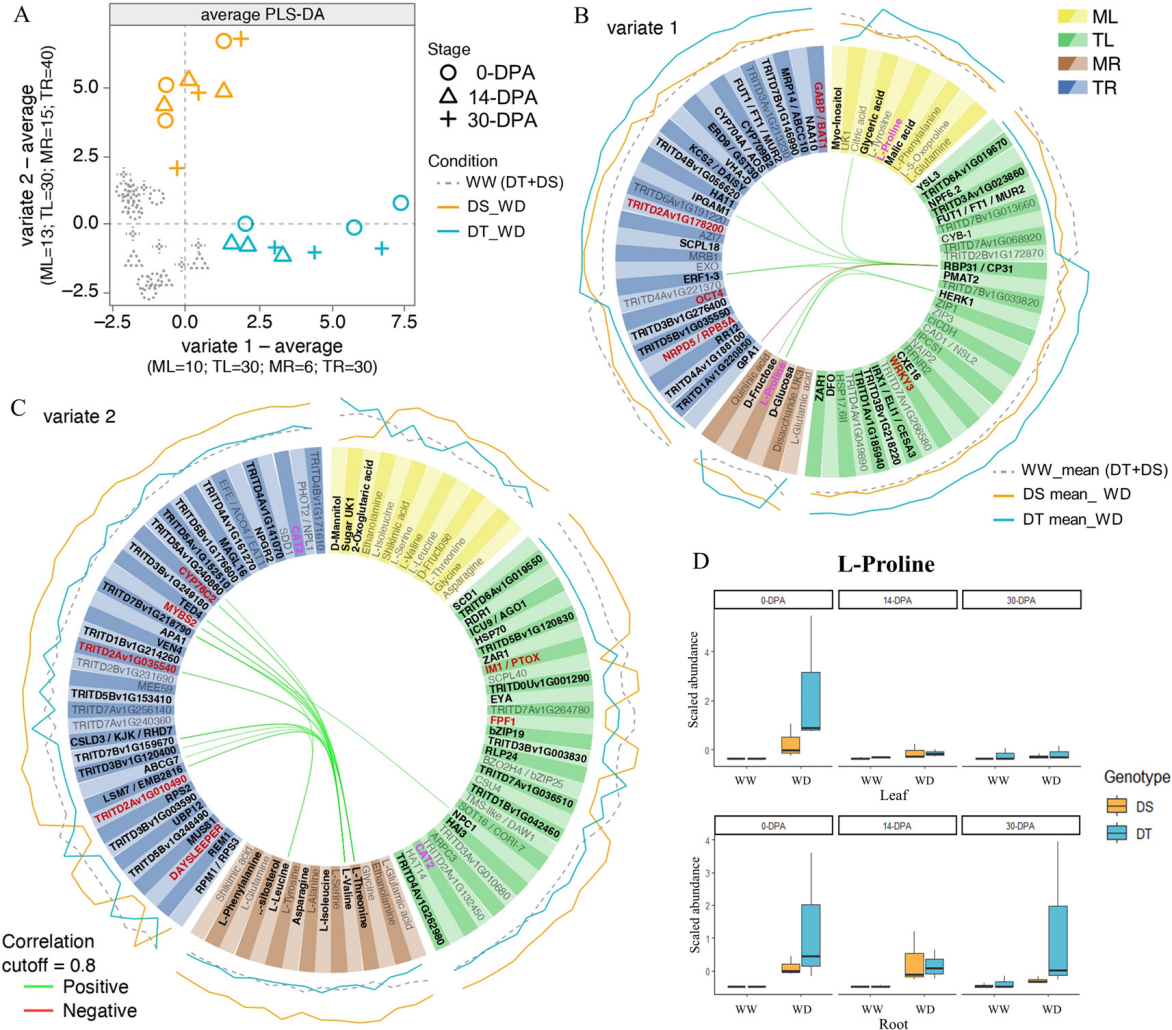
Leaf and root transcriptomic and metabolomic integration analysis of durum wheat considering two water treatments (WW and WD) at three developmental stages (0-, 14-, and 30-DPA) in susceptible (DS) and tolerant (DT) genotypes. **(A)** Average sparse partial least square discriminant Analysis (sPLS-DA) with selected variables using leaf metabolomic (ML), leaf transcriptomic (TL), root metabolomic (MR), and root transcriptomic (TR) as omic datasets for integration. Circosplots showing the root and leaf selected metabolites and transcripts for the DT genotype (variate 1; **B**) and DS genotype (variate 2; **C**). The expression values of each transcript are plotted on the outside part of the circosplots, considering the average value of all replicates for each condition (WW-mean, DS-WD-mean, and DT-WD-mean). Positive and negative correlations between omic data sets were represented by green and red lines in the center, respectively. In red, transcripts were located on chromosome 2A, where constitutive QTLs were identified. **(D)** L-Proline abundance in tolerant (DT) and susceptible (DS) genotypes in leaf and root under well-water and water deficit conditions.

To integrate transcriptomic and metabolomic data and find the lowest number of variables that allow explaining the separation between the DT and the DS genotypes under WD conditions and both genotypes under WW conditions, a sPLS-DA was performed using each sampling time as replicas (**Figure 5**). The number of DEGs used for the analysis was obtained from the union of a total of DEGs identified that respond to drought in both genotypes and a total of DEGs identified due to a differential response between genotypes. Therefore, the total list of DEGs was 697 and 900 DEGs for leaf and root, respectively. A clear separation was observed between the genotypes under WW condition and the DT genotype under the WD condition with variate 1. In contrast, a separation was observed between the genotypes under WW condition and the DS genotype under the WD condition with variate 2 (**Figure 5A**). Considering a two-variate analysis, the tuning function was performed to determine the optimal number of variables to select for the leaf and root dataset. For variable 1, which separates the DT genotype, 60 (30 in root and 30 in leaf) transcripts and 16 (6 in root and 10 in leaf) metabolites that best explained the separation were selected (**Figure 5B**), and their expression/abundance is shown in **Supplementary Table S20**. Regarding variable 2, which separates the DS genotype, 70 (40 in root and 30 in leaf) transcripts and 28 (15 in root and 13 in leaf) metabolites were selected (**Figure 5C**), and their expression/abundance is shown in **Supplementary Table S21**. A circosPlot with the correlations between the selected transcripts and the metabolites for each variate in the DT and DS genotypes is shown in **Figures 5B, C**. Finally, L-Proline was an important metabolite in both tissues (leaf and root) that allowed differentiating the tolerant genotype from the susceptible one (**Figure 5D**), suggesting it as a good biomarker.

To identify possible biomarkers associated with drought tolerance/susceptibility in durum wheat, the variables that exhibited a consistent differential expression/abundance across the three sampling times in each genotype were determined. Regarding the transcriptomic data, 15 and 24 genes had higher differential expression in leaf and roots, respectively. Interestingly, the TRITD6Av1G194890 gene that encodes a Galactoside 2-alpha-L-fucosyltransferase and the L-proline metabolite were significant in both tissues in the DT genotype. In addition, the metabolites such as malic acid, glyceric acid, Myo-inositol, and L−proline were appropriate candidates for leaf, and the sugars such as D-fructose and D-glucose and the amino acid L-proline were appropriate for roots. These results confirm that the L-proline is a very good candidate as a biomarker of tolerance to drought, independent of tissue and developmental stages. In addition, our study allowed us to determine new tissue-specific biomarkers in durum wheat. The catalase was an interesting gene in the DS genotype that responds to drought stress in both tissues. Therefore, it could be a proper biomarker of susceptibility to drought in durum wheat. The metabolites such as 2-oxoglutaric acid, D-mannitol, and an unknown sugar were adequate candidates for leaf. The amino acids such as L-phenylalanine, L-leucine, asparagine, L-isoleucine, L-valine, L-threonine, and b-sitosterol were suitable for roots.

## 4 Discussion

### 4.1 Genetic dissection of drought tolerance in durum wheat

In recent decades, climate change has increased drought frequency, duration, and extent during plant development, causing significant grain yield losses worldwide and affecting food security (Bailey-Serres et al., 2019). Uncertainties about the future effects of climate change require breeders to develop varieties that perform well in diverse environments, which is challenging since broad adaptation is hampered by high genotype-environment interactions for most yield-related traits (Zaïm et al., 2024). The ANOVA analysis across eight environments showed a larger contribution of the G×E interaction to the total variability compared to the G effect for all traits except for TGW, which agrees with their heritability as reported in previous studies (Brinton and Uauy, 2019; Arriagada et al., 2020). Thus, developing high-yielding varieties adapted to diverse environments requires the accumulation of loci with additive genetic effects on yield components. In this sense, numerous studies have been carried out to identify quantitative trait loci through QTL or associative mapping related to the main yield components under irrigated and rainfed conditions in durum wheat (Sukumaran et al., 2018; Maccaferri et al., 2019; Arriagada et al., 2022; Zaïm et al., 2024). Most common wheat loci have been localized on chromosomes 2B, 3A, 4A, 4B, 7A, and 7B (Gupta et al., 2017; Goel et al., 2020). In durum wheat, the most significant MTAs associated with yield components under irrigated, rainfed, and heat stress were identified on chromosomes 2A and 2B (Sukumaran et al., 2018). This agrees with our results, where the chromosomes with the highest number of MTAs were 2A (351), 2B (102), and 7B (53), and the traits most reported were TGW and GPS (Zaïm et al., 2024).

The QTLs identified across different environments (stable QTLs) can be considered robust candidates for improving drought tolerance and increasing grain yield (Soriano et al., 2021; Arriagada et al., 2022). This study identified nine constitutive MTAs across eight environments under rainfed and irrigated conditions. These MTAs were grouped into three QTLs associated with TGW and GPS, two QTLs located on chromosomes 2A (QTN_2A_TGW/GPS.1; QTN_2A_TGW/GPS.2) and one on chromosome 2B (QTN_2B_TGW/GPS.1), explaining between 5.15% and 14.29% of the variation. In a previous Meta-QTL (MQTL) analysis, Arriagada et al. (2022) identified three MQTL associated with yield components on chromosomes 2A (2) at 71.4 and 88.7 cM and 2B (1) at 28.9 cM associated with TGW and HI under rainfed and irrigated conditions. Moreover, Soriano et al. (2021) identified an MQTL on chromosome 2A at 50.8 cM related to normalized difference vegetation index (NDVI) and chlorophyll content (SPAD), traits that are associated with grain yield under drought stress. Sukumaran et al. (2018) also identified a stable QTL under irrigation and drought conditions on chromosome 2A at 66–70 cM associated with TGW. These results suggest that regions on chromosomes 2A and 2B are useful in durum wheat breeding programs, in which the goal is to develop varieties adapted to different environments with high grain yields.

### 4.2 Physiological response to drought stress in durum wheat

Reductions of 1% in net photosynthesis (Pn), 8% in intercellular carbon concentration (Ci), and 21% in transpiration (T) were observed in the water deficit (WD) treatment, with stomatal conductance (gs) 9% lower across genotypes. Zhao et al. (2020) reported a 20% decrease in Pn in bread wheat under moderate deficit irrigation, while Bogale et al. (2011) noted significant reductions in Pn and T in durum wheat under WD conditions, aligning with earlier findings (Condon et al., 2002; Allahverdiyev, 2015). Measurements in this study were taken two weeks after starting the WD treatment, and gas exchange and yield components response vary based on the frequency, intensity of water stress, and wheat growth stage and may not have been reflected to a full extent.

The G3 genotype exhibited significant susceptibility to water deficit (WD), with all gas exchange parameters declining and lower NBI and Chlorophyll Content (CC). In contrast, G4 genotype showed a 66% reduction in gs and a 61% decrease in T but maintained stable Ci and Pn and higher NBI and CC, linked to the chromosome 2 findings previously discussed (Sukumaran et al., 2018; Soriano et al., 2021; Zaïm et al., 2024). Pn reduction is primarily due to stomatal closure under water stress, linked to increased ABA in the xylem (Liu et al., 2015). Still, non-stomatal limitations also arise from lowered enzymatic efficiency or activation, including RuBP carboxylation and regeneration (Flexas and Medrano, 2002). Water stress can also damage the oxygen-evolving complex and PSII reaction centers (Sainz et al., 2010). Severe drought slows ATP renewal as proton concentration rises in the thylakoid lumen, leading to more PSII heat dissipation. Research indicates that Rubisco activity and RuBP reduction do not limit Pn until drought becomes severe (Boutraa et al., 2010). Also, gas exchange parameters may not change significantly until the volumetric water content of the soil decreases by more than two-thirds (Sinclair, 2005).

### 4.3 Transcriptomics response to drought in durum wheat

The stress signaling mechanism and its regulatory networks that control the expression of critical genes are the basis of plant tolerance to drought (Janiak et al., 2016). In general, it was observed that the response to drought stress in durum wheat acted in a tissue- and time-specific manner since neither the number of DEGs nor their functional annotation was standard between leaf and root in the different sampling times studied in this work. There was a higher amount of DEGs in the root compared to the leaf for comparisons between genotypes and treatments at 0-, 14- and 30- DPA. Moreover, most drought response genes were regulated at 0-DPA in both genotypes, where the number of DEGs in the DT genotype (1,174) was much higher than in the DS genotype (84). This result is expected since the plant responds to drought stress by sensing the water shortage in the soil through its roots (Janiak et al., 2016). Then, a signaling cascade is activated that sends chemical signals to the tissues of the aerial part, such as shoots and leaves (Kuromori et al., 2022), to initiate a response that leads to physiological, biochemical, and morphological changes that allow plants to tolerate stress conditions (Janiak et al., 2016).

Drought stress modulates molecular responses associated with the biosynthesis of antioxidants, photosystem components, ABA metabolism, biosynthesis of secondary metabolites, and metabolism of carbohydrates and tricarboxylic acids in durum wheat (Zuluaga et al., 2023). This study’s GO analysis showed genotype-, time-, and tissue-specific results, revealing that some important biological processes occur differently in the two contrasting genotypes [G3 (BRESCIA, drought-susceptible) and G4 (QUC 3678-2016, drought-tolerant)] evaluated under water stress. However, some biological processes were common, including cell death, response to salicylic acid, and biosynthesis of secondary metabolites. Under drought stress, the salicylic acid (SA) phytohormone regulates the antioxidant defense system, transpiration and photosynthesis rates, nutrient uptake, stomatal movement, nitrogen metabolism, cell elongation, proline metabolism, inhibiting lipid oxidation, and deactivating reactive oxygen species (ROS) (Khan et al., 2015; Khalvandi et al., 2021; González-Villagra et al., 2022). In wheat, the SA induced responses against drought by increasing antioxidative enzymes and osmolytes such as proline and total soluble sugars (Sharma et al., 2017; Khalvandi et al., 2021).

Additionally, plants with exogenous applications of SA have higher biomass, higher chlorophyll content, higher water content, higher Rubisco carboxylase activity, and higher superoxide dismutase (SOD) activity relative to untreated plants (Singh and Usha, 2003). On the other hand, plants synthesize secondary metabolites such as flavonoids, spermidine, and plant hormones to scavenge ROS and protect themselves from lipid peroxidation when subjected to adverse environmental conditions (Yadav et al., 2021). Direct regulation of genes in the biosynthetic pathway of secondary metabolites or indirect regulation through phytohormone regulation provides the plant with drought tolerance by avoiding oxidative stress (Jogawat et al., 2021). Therefore, phytohormones such as salicylic acid and secondary metabolites play a key role in the drought tolerance of durum wheat. Finally, a recent study indicates that post-anthesis drought stress greatly influences wheat grain development and programmed cell death in the endosperm. The expression of genes related to the glutathione-ascorbic acid cycle pathway (DNA repair) and the mitogen-activated protein kinase (MAPK) signaling cascade pathway (membrane functions) were key pathways involved in regulating the programmed cell death at two key stages of endosperm development, 7 days and 14 days post anthesis (Li et al., 2024), which agrees with our results.

At the gene level, four DEGs showed a constitutive response to drought stress from 0- to 30-DPA in leaves of the DT genotype. However, only two genes (TRITD2Bv1G146530 and TRITD4Bv1G179760) were up-regulated all three times sampled. The TRITD2Bv1G146530 gene located on chromosome 2B (434782763–434784101 bp) encodes a plastid-localized arogenate dehydratase (ADT) involved in phenylalanine biosynthesis. The amino acid phenylalanine serves as a building block for proteins and as a main precursor for the biosynthesis of phenylpropanoids that play a key role in plant growth, development, and the response to environmental signals (El-Azaz et al., 2020). In *Arabidopsis*, the arogenate dehydratase gene (ADT2) plays an essential role in seed development because an ADT2 deficiency causes seed abortion (El-Azaz et al., 2018), which in wheat can translate into a loss of grain yield. In common wheat, the arogenate dehydratase 5 (ADT5) expression was increased in leaf tissues of a sensitive and tolerant cultivar after 4 h drought stress. However, the expression level of ADT5 in the leaf was increased by eight-fold and decreased by two-fold in the tolerant and susceptible cultivars, respectively, after 8 h of drought stress (Cevher-Keskin et al., 2023). The TRITD4Bv1G179760 gene on chromosome 4B (607000032 – 607006–701 bp) encodes a chloroplast RNA-binding protein (RBP). The RBPs govern many aspects of RNA metabolism, including pre-mRNA processing, transport, stability/decay, and translation, and are also involved in regulating the response to abiotic stresses (Ambrosone et al., 2012; Muthusamy et al., 2021; Yan et al., 2022), including drought in wheat (Xu et al., 2014; Sun et al., 2020). Transgenic plants were generated in rice to investigate the potential use of RBPs in the development of drought-tolerant cultivars. The expression of the Arabidopsis glycine-rich RNA-binding protein (AtGRP2 or AtGRP7) caused an increase in the number of grains per panicle, therefore increasing grain yield under drought conditions (Yang et al., 2014). Our results show that the ADT and RBP genes were up-regulated at 0-, 14-, and 30 DPA in the DT genotype under WD conditions, indicating that these genes can be keys in the drought response in durum wheat, which has not been previously reported.

In root, 11 DEGs were constitutive in the DT genotypes under WD conditions (**Supplementary Table S22**). Among them, three genes were highly up-regulated (Log2FC > 30). The TRITD1Av1G135510 gene encodes a bifunctional inhibitor/lipid-transfer protein/seed storage 2S albumin superfamily protein related to the lipid metabolic process (Quinga et al., 2018). This gene was up-regulated under osmotic and drought stress in barley (Paul et al., 2023) and wheat (Pál et al., 2018), respectively. In addition, it is highly up-regulated in phellem cells, participating in suberization and/or secondary cell wall development in the roots of *Arabidopsis* (Leal et al., 2022). The TRITD2Bv1G203390 gene that encodes a proline-rich protein DC2.15. The proline-rich proteins (PRP) are stress-induced and are involved in cell wall modification and organization (Fan et al., 2014). In a transcriptomic analysis of DEGs in two alfalfa cultivars with different salt tolerance, the MS.gene011517 (14 kDa proline-rich protein DC2.15) had higher and consistent expression under salt stress (Bhattarai et al., 2021). In addition, PRP may regulate free cellular proline levels during drought stress to provide drought tolerance in tomatoes (Gujjar et al., 2018). Finally, the TRITD4Av1G258040 gene that encodes a Serine/threonine-protein kinase (S/T PK) was also highly up-regulated in the root of durum wheat. Protein kinases are essential enzymes that regulate many metabolic pathways through protein phosphorylation/dephosphorylation to regulate cellular functions such as signal transduction and response to abiotic stress (Mao et al., 2010). The S/T PK is important in maintaining root morphology during stress conditions (Muhammad et al., 2019). In wheat, several S/T PKs involved in drought tolerance have been characterized, such as *TaSnRK2.7* (Zhang et al., 2011), *TaSnRK2.4* (Mao et al., 2010), PK4 (Rahimi et al., 2021), RPK1 (Rahim et al., 2022). In durum wheat, the only reported and characterized gene that encodes for an S/T PK and is associated with drought tolerance is *Td4IN2* (Rampino et al., 2017).

### 4.4 Metabolic response to drought in durum wheat

To explore the metabolic response of durum wheat grown under drought, GC–MS in leaf and root tissues determined polar metabolites relative abundances. Metabolites play a fundamental role in drought tolerance since they are considered signaling molecules (Obata and Fernie, 2012). Overall, 43 metabolites were identified and quantified, most of them were amino acids, sugars, and organic acids. This result agrees with other metabolic studies in wheat under drought stress conditions, where the most abundant identified metabolites are amino acids, organic acids, and sugars (Solomon and Labuschagne, 2005; Guo et al., 2018; Marček et al., 2019; Fadoul et al., 2021). Amino acids are essential plant metabolites for protein synthesis and cellular function (Ahanger et al., 2018). Free amino acids act as osmoprotectants and improve drought-induced nitrogen uptake through reassimilation and maintaining protein homeostasis (Živanović et al., 2020). In addition, they also function as scavengers of reactive oxygen species (ROS) generated in plants under drought conditions (Kumar et al., 2021). Organic acids are important in energy metabolism because they are involved in the oxidative and synthetic pathways of carbohydrates and fatty acids (Fadoul et al., 2021). Besides this, they are precursors of amino acids and increase the osmotic potential of the cell, which implies an increased water-holding capacity (Martinelli et al., 2013; Zeng et al., 2021). Finally, sugars play an important role in plant metabolism as sources of carbon and energy in cells, the maintenance of osmotic balance, as signaling molecules for photosynthesis, and detoxification of ROS by acting as metabolic signals in stress conditions (Li C. et al., 2016; Ahmad et al., 2020).

Five metabolites showed differential abundance across 0- and 30-DPA when genotypes under WD conditions in the leaf were compared. These metabolites were D-mannitol, asparagine, aspartic acid, L-glutamic acid, and malic acid. The amino acid asparagine is central to nitrogen storage and transport due to its relatively high nitrogen-to-carbon ratio and non-reactive nature (Curtis et al., 2018). Further, it accumulates in high concentrations during processes such as seed germination and in response to various abiotic stresses due to its potential role as an osmolyte (Carillo et al., 2005; Lea et al., 2007). In this study, asparagine levels were higher in the DS than in the DT genotype under drought stress, consistent with rice observations (Degenkolbe et al., 2013) and wheat (Yadav et al., 2019). The asparagine accumulation may be associated with a biochemical response to dehydration, likely senescence, that has negative consequences for yield (Yadav et al., 2019), in agreement with our results where senescence was an enriched process in the transcriptomic analysis comparing genotypes under WD conditions. Glutamic acid participates in different biological processes, playing an important role in the biosynthesis of chlorophyll and proline (Franzoni et al., 2021). It has been shown that the exogenous application of glutathione protected wheat seedlings from water deficit damage, increasing chlorophyll pigments and antioxidant enzyme activity and promoting the root system, shoot growth, and relative leaf water content (Suliman et al., 2024). Moreover, the overexpression of the cytosolic glutamine synthetases (GS) 1 and plastidic GS2 genes from wheat enhanced drought tolerance of both root and leaf tissues in tobacco (Yu et al., 2020). At 0-DPA, levels of L-glutamic acid were higher in the DS than in the DT genotype. However, at 14- and 30-DPA the levels were higher in DT than in the DS genotypes. Therefore, the higher proline contents in the DT genotype could indicate the greater tolerance to drought stress, induced by glutamic acid.

In root, four DAMs were constitutive in the comparison between contrasting genotypes under WD conditions: organic acids (aconitic acid, aspartic acid, and glyceric acid) and L-Valine. Aconitic acid is an intermediate in the glyoxylate and tricarboxylic acid (TCA) cycles produced during the conversion of citric acid to isocitrate (Min et al., 2018). In wheat, its positive regulation increases energy for the defense against oxidative stress (Liu et al., 2020). In this study, aconitic acid levels were higher in the DS genotype across the three developmental stages under WD conditions. However, the aconitic acid levels decreased significantly after 0-DPA in DS genotype with drought stress, which agrees with previous findings in common wheat (Guo et al., 2018). Aspartic acid is obtained from a transamination reaction between glutamate and oxaloacetate, which then produces the amino acids lysine, threonine, methionine and isoleucine, in a series of reactions known as the aspartic acid metabolic pathway (Alfosea-Simón et al., 2021). In common wheat, exogenous application of aspartic acid has alleviated salt stress-induced growth decline by enhancing antioxidants, compatible solutes, and reducing reactive oxygen species (Sadak et al., 2022). Finally, Weng et al. (2024) showed that eight metabolites (L-tryptophan, L-valine, L-leucine, glycerol 1-phosphate, L-threonine, gluconic acid lactone, malonic acid, D-malic acid) were increased and two (quinic acid, aspartic acid) were decreased constitutively in a DT genotype of common wheat under drought stress conditions, similar as it was found in this study.

### 4.5 Integration of transcriptomic and metabolomic data

Tolerance to drought involves activating a series of complex mechanisms by the plant. This begins with the detection of stress, which activates a signal transduction pathway that activates stress response genes that are translated into proteins and compounds whose main function is to prevent water loss in the cells. Therefore, the integration of multi-omics data from genomics, transcriptomics, proteomics and metabolomics analysis allows to obtain a comprehensive understanding of these complex biological processes and to identify the key molecular players involved in the drought response of the plant (Roychowdhury et al., 2023). In this sense, to identify possible key biomarkers associated with drought tolerance/susceptibility in durum wheat, the variables that showed consistent differential expression/abundance across 0- to 30-DPA in each genotype were determined.

Regarding the transcriptomic data of the DT genotype on leaf, the TRITD2Av1G062130 gene located on chromosome 2A (140,485,202-140,488,307 bp) that encodes a WRKY transcription factor (WRKY3) is positioned on the stable QTL “QTN_2A_TGW/GPS.1”, whose SNP marker in LD is GENE-1342_238. The WRKY proteins constitute a large family of transcription factors involved in the regulation of development, senescence and stress tolerance, including drought (Zhu et al., 2013; Wang et al., 2023). Several studies in wheat have shown that overexpression of WRKY transcription factors such as TaWRKY1, TaWRKY10, TaWRKY33, TaWRKY40, TaWRKY75 enhances drought tolerance (Yu et al., 2023). In root, four identified genes were located on chromosomes 2A and 2B, where stable QTLs were located. The TRITD2Av1G187060 (Amino acid permease), TRITD2Av1G178200 (Uridylate kinase), TRITD2Av1G265880 (Organic cation transporter protein), and TRITD2Bv1G172660 (DNA-directed RNA polymerase II, putative) genes, however, none of them was present within the regions of the detected stable QTLs. Interestingly, TRITD6Av1G194890 gene that encodes a Galactoside 2-alpha-L-fucosyltransferase was important in both tissues in the DT genotype, in agreement with studies showing that this enzyme participates in drought tolerance (Li S. et al., 2016; Moradi Tarnabi et al., 2020; Sui and Wang, 2020). Regarding metabolites in the DT genotype, the malic acid, glyceric acid, myo-Inositol, and L−proline were good candidates for leaf, and the sugars such as D-fructose and D-glucose and the amino acid L-proline were appropriate for roots. This result confirms that the L-proline is an outstanding candidate as biomarker for drought tolerance, independent of tissue and developmental stages. Proline accumulation functions as an electron sink mechanism that can reduce the amount of singlet oxygen present due to drought stress, contributing to cellular redox balance (Ullah et al., 2017). In the DS genotype, the catalase (CAT2) was an interesting gene that responds to drought stress in both tissues. Therefore, it could be a promising biomarker of susceptibility to drought in durum wheat. The *CAT* gene expression regulates growth, development, and response to environmental stress (Zhang et al., 2022). The *CAT* gene family encodes an antioxidant enzyme that modulates the hydrogen peroxide (H_2_O_2_) contents in cells by translating this toxic compound into water (H_2_O) and O^2−^ to reduce ROS contents in cells, produced because of drought stress (Ghorbel et al., 2023). Finally, the metabolites such as 2-oxoglutaric acid, D-mannitol and an unknown sugar were good candidates for leaf, and the amino acids such as L-phenylalanine, L-leucine, asparagine, L-isoleucine, L-valine, and L-threonine, and the b-Sitosterol were appropriate for roots.

To our knowledge, this is the first study evaluating different omics approaches and their integration to decipher drought tolerance in durum wheat. The genomic approach identified nine stable QTLs under rainfed and irrigated conditions across eight environments. These QTLs were located on chromosomes 2A and 2B and associated with TGW and GPS traits, suggesting that these regions may be useful in durum wheat breeding programs, where the goal is to develop varieties adapted to different environments with a high yield. Through independent transcriptomic and metabolomic analysis, it was possible to identify genes and metabolites that show differential expression/abundance across the different developmental stages (0 to 30 DPA) in the drought-tolerant genotype of durum wheat. Finally, by integrating omics, a set of leaf- and root-specific genes and metabolites were identified, allowing differentiation between the tolerant and susceptible genotypes of durum wheat. This result permits the determination of new tissue-specific biomarkers for drought-stress responses in durum wheat through their characterization and validation in future studies. These results are valuable and novel in durum wheat breeding programs for developing resilient and high-yielding cultivars to ensure food security.

## Data Availability

The original contributions presented in the study are publicly available. This data can be found here:NCBI, PRJNA1261096.
