# Supplementary material for: Combined multi-omics and physiological approaches to elucidate drought-response mechanisms of durum wheat
Source: Front Plant Sci. 2025 May 15;16:1540179. doi: 10.3389/fpls.2025.1540179 (PMC12119503; doi:10.3389/fpls.2025.1540179)
Supplement: Supplementary file 1 [file DataSheet2.docx]

Supplementary Material

## Supplementary Figures


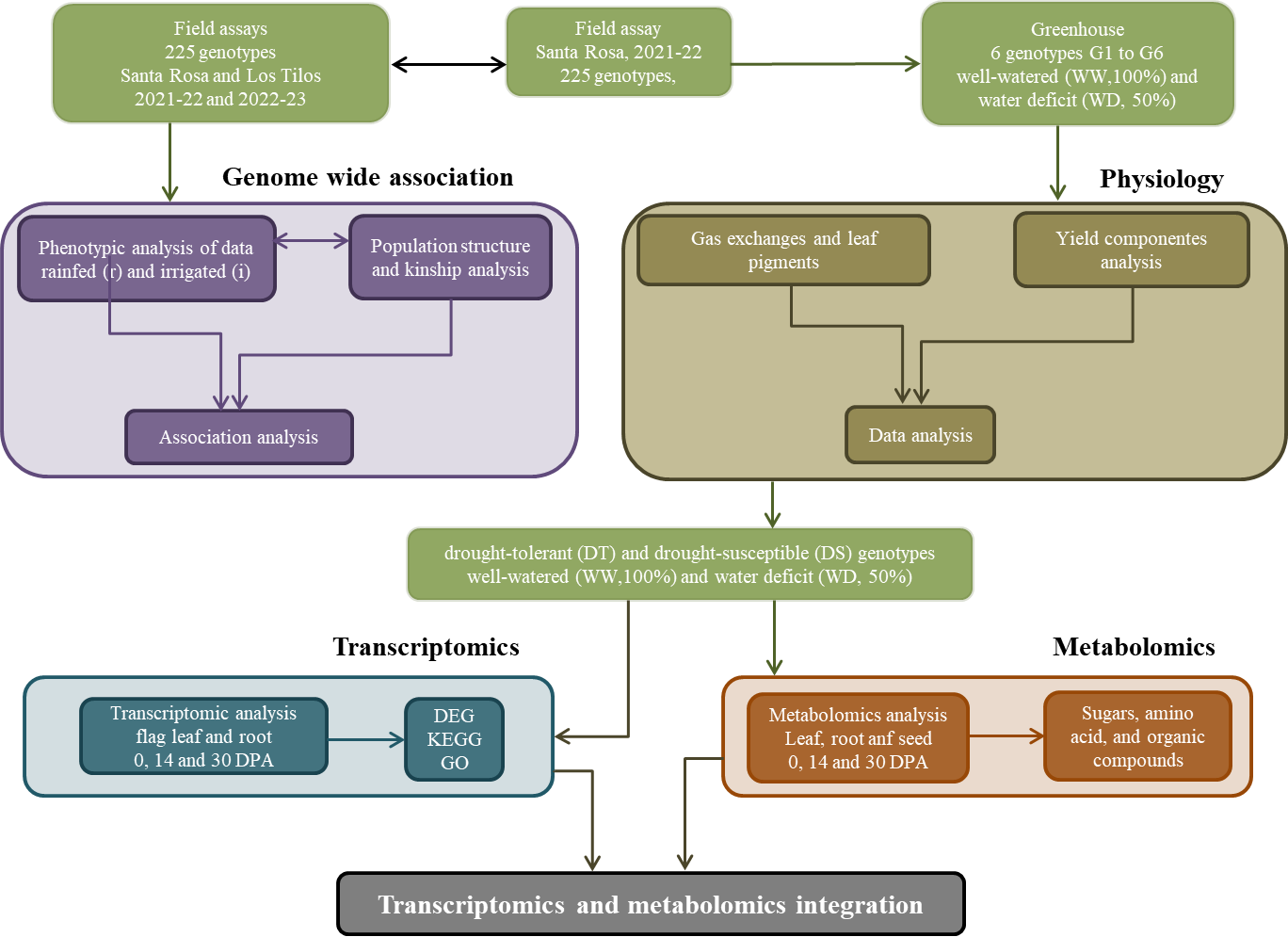


**Supplementary Figure 1.**Experimental setup summary of this work.


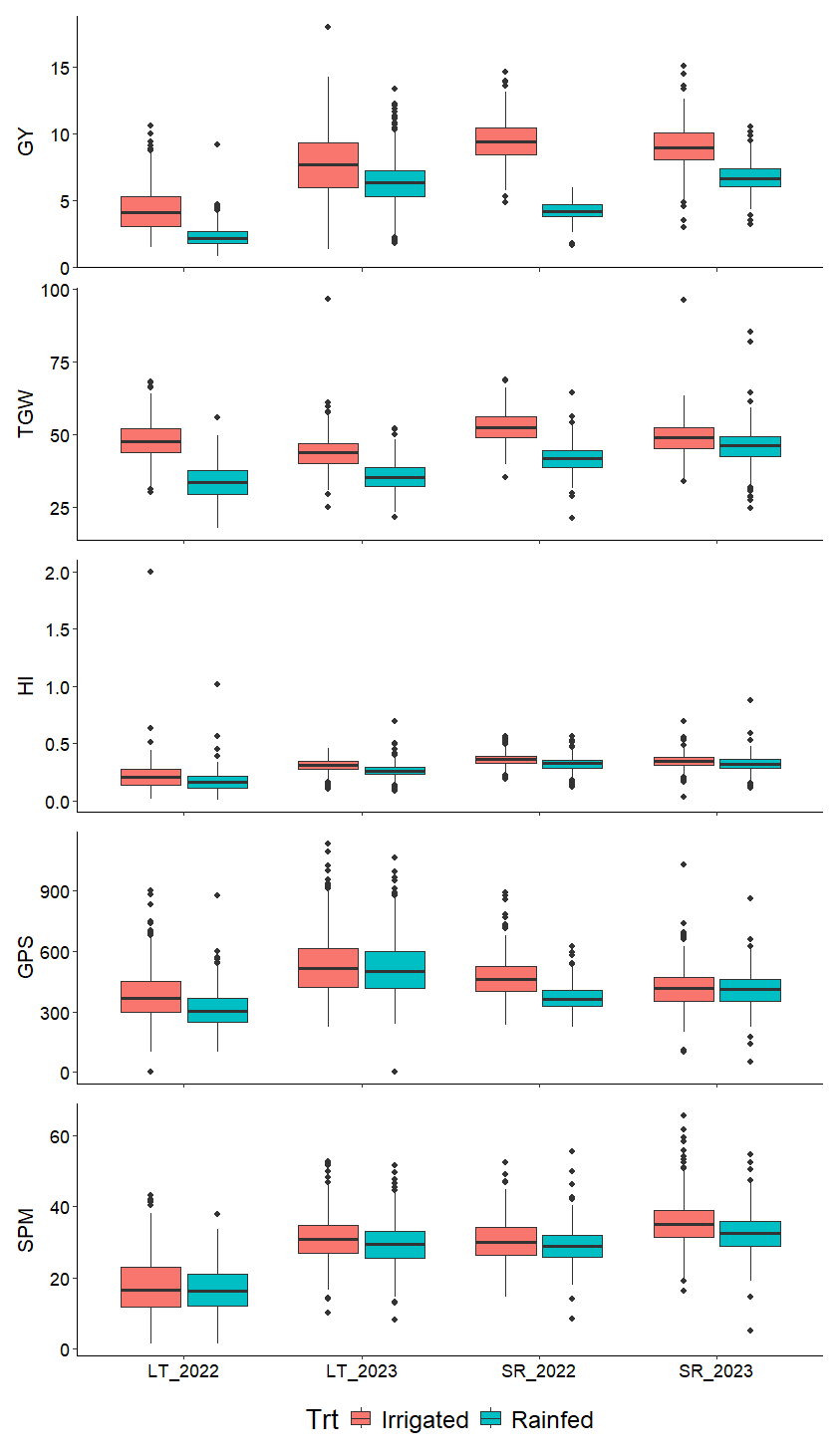


**Figure S2**. Boxplots of yield-related traits of 225 durum wheat genotypes across 8 environments under irrigated and rainfed conditions. The medians are indicated by black line inside the boxes. The box borders indicate upper and lower quartiles, the caps indicate 90^th^ and 10^th^ percentiles, and the circles indicate observations below and above those percentiles. GY: grain yield (t ha^-1^); TGW: 1,000-grain weight; HI: harvest index; GPS: grain per spike; SPM: spike per m^2^. Locations LT: Los Tilos; SR: Santa Rosa; Growing seasons 2022: 2021/22; 2023: 2022/23.


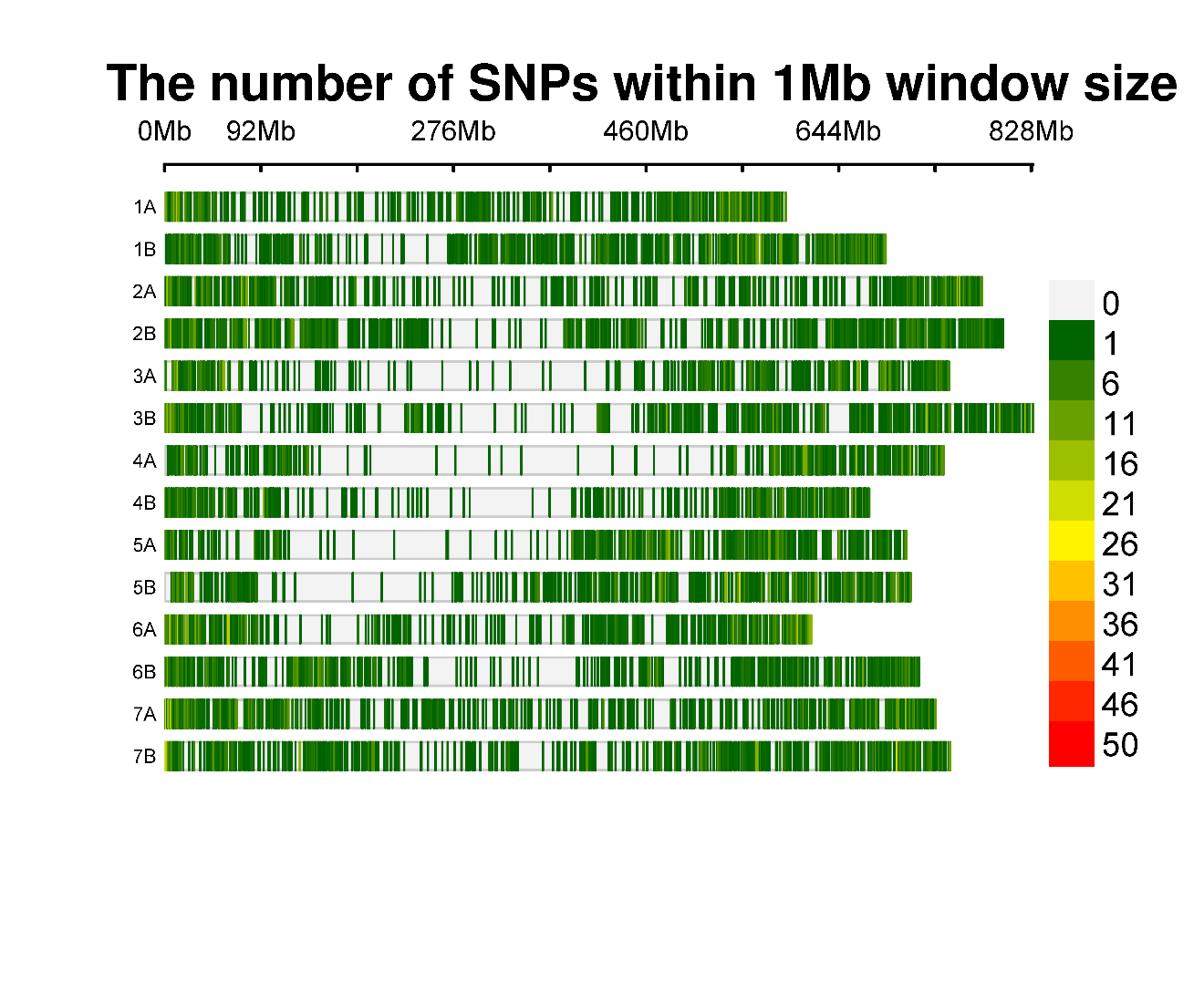


**Figure S3**. Distribution of the 12,498 SNP markers on the 14 chromosomes of durum wheat. Colored bars are SNP counts within 10 Mb window size.


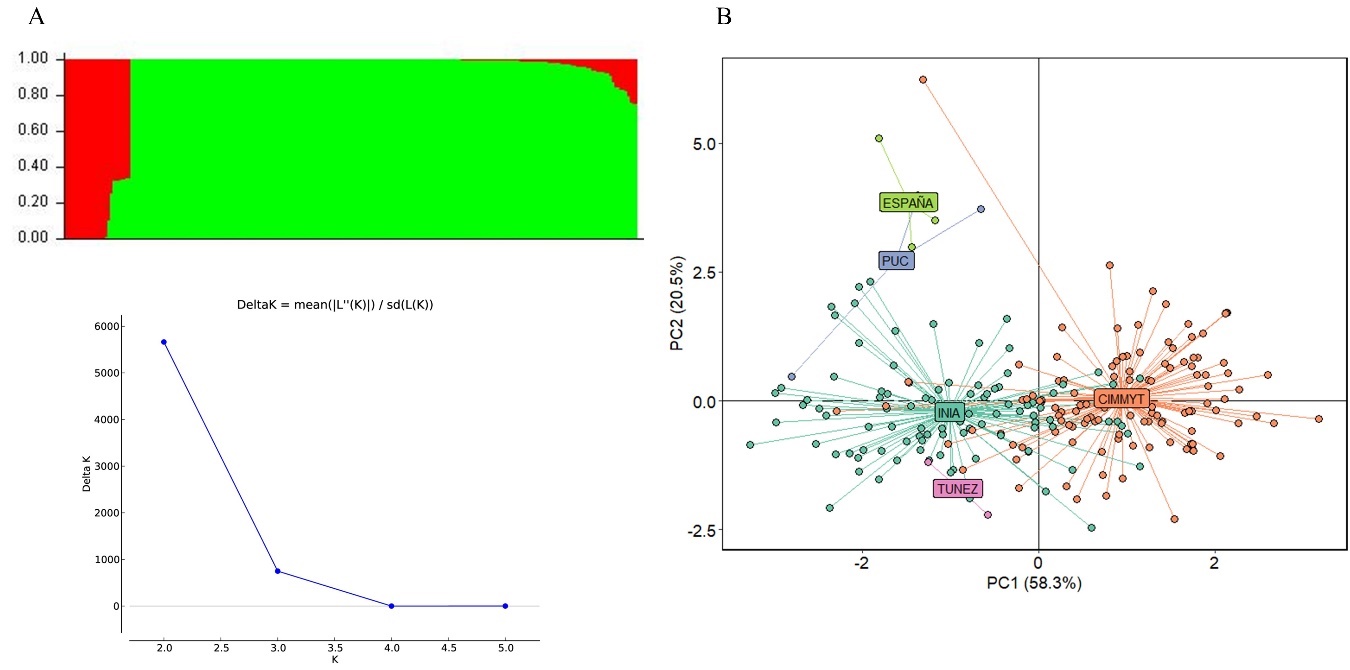


**Figure S4**. Population structure of the 245 durum wheat genotypes based on 12,498 SNP markers. (A) Structure analysis; (B) Delta K for subpopulations numbers; and (C) Principal components analyses DAPC showing the clustering among genotypes origin. Abbreviations: CIMMYT - International Maize and Wheat Improvement Center, Mexico; INIA - The Institute of Agricultural Research, Chile; PUC - Pontifical Catholic University of Chile; IRTA - Institute of Agrifood Research and Technology, Spain; and INRAT - National Institute of Agronomic Research, Tunisia.

**
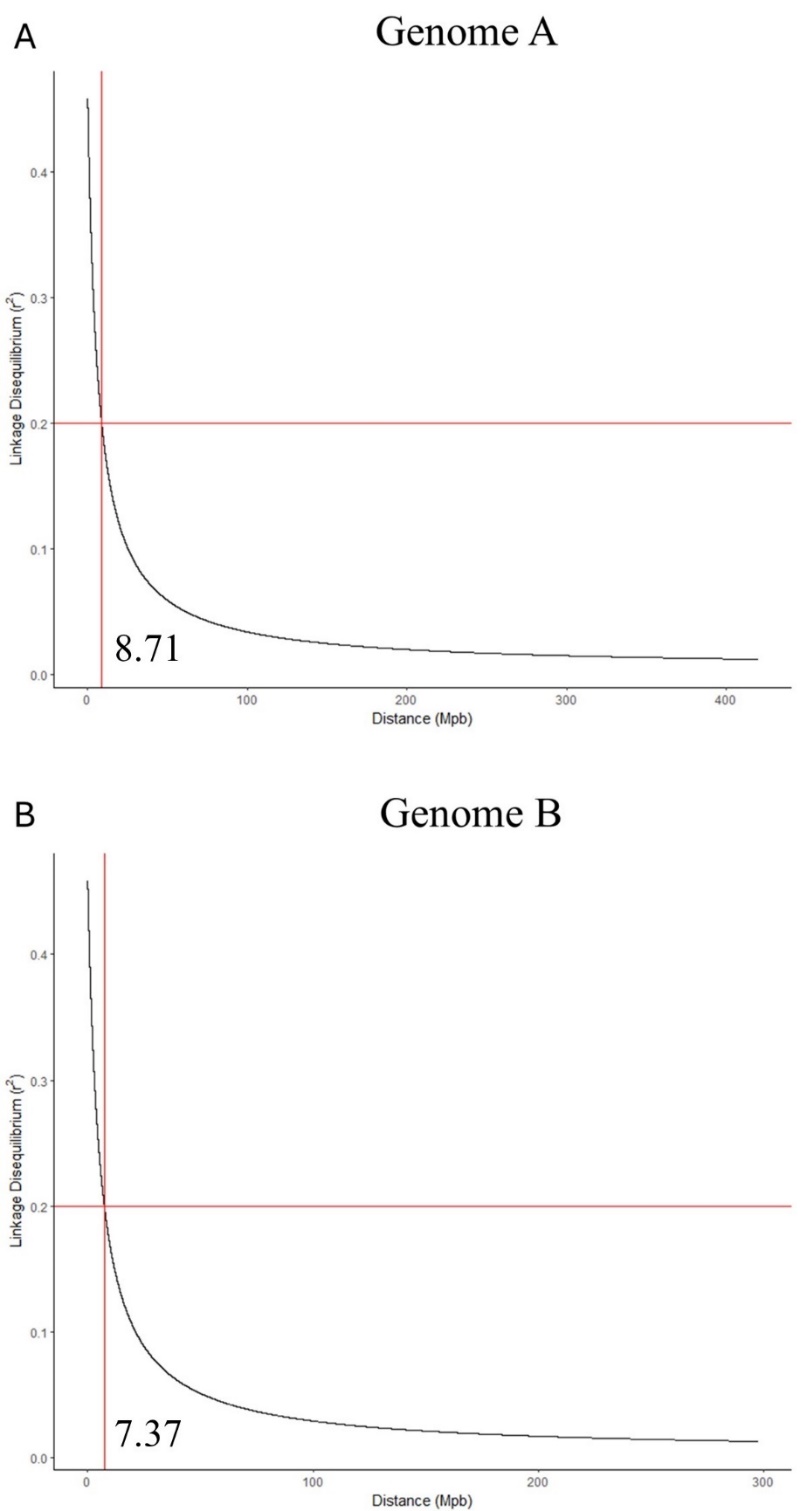
**

**Figure S5**. Linkage disequilibrium (LD) decay showing approximate distances of 8.71 Mpb and 7.37 Mbp on genome A and B of durum wheat, respectively.


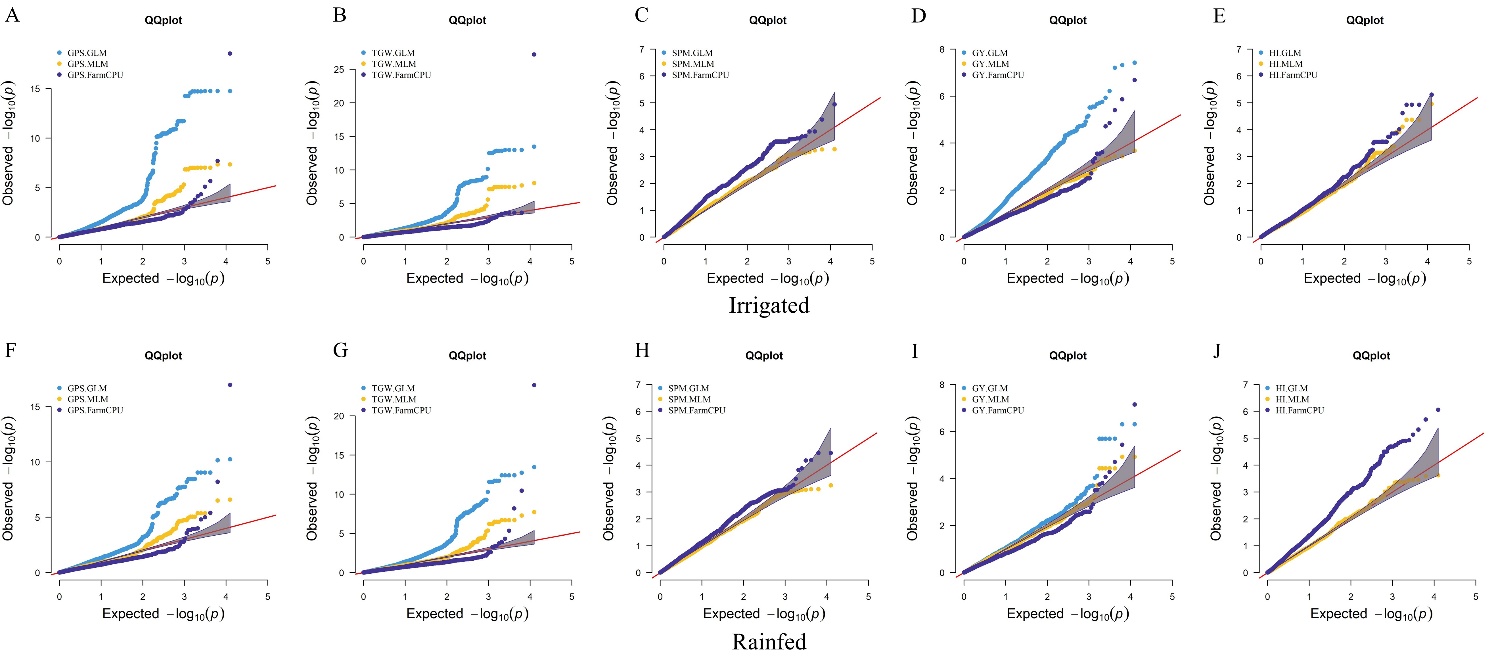


**Figure S6.** Quantile-quantile (QQ) plot of the general linear model (GLM), mixed linear model (MLM), and fixed and random circulating probability unitization model (FarmCPU) for the GPS, TGW, SPM, GY, and HI under irrigated (A, B, C, D, and E) and rainfed (F, G, H, I, J) conditions.


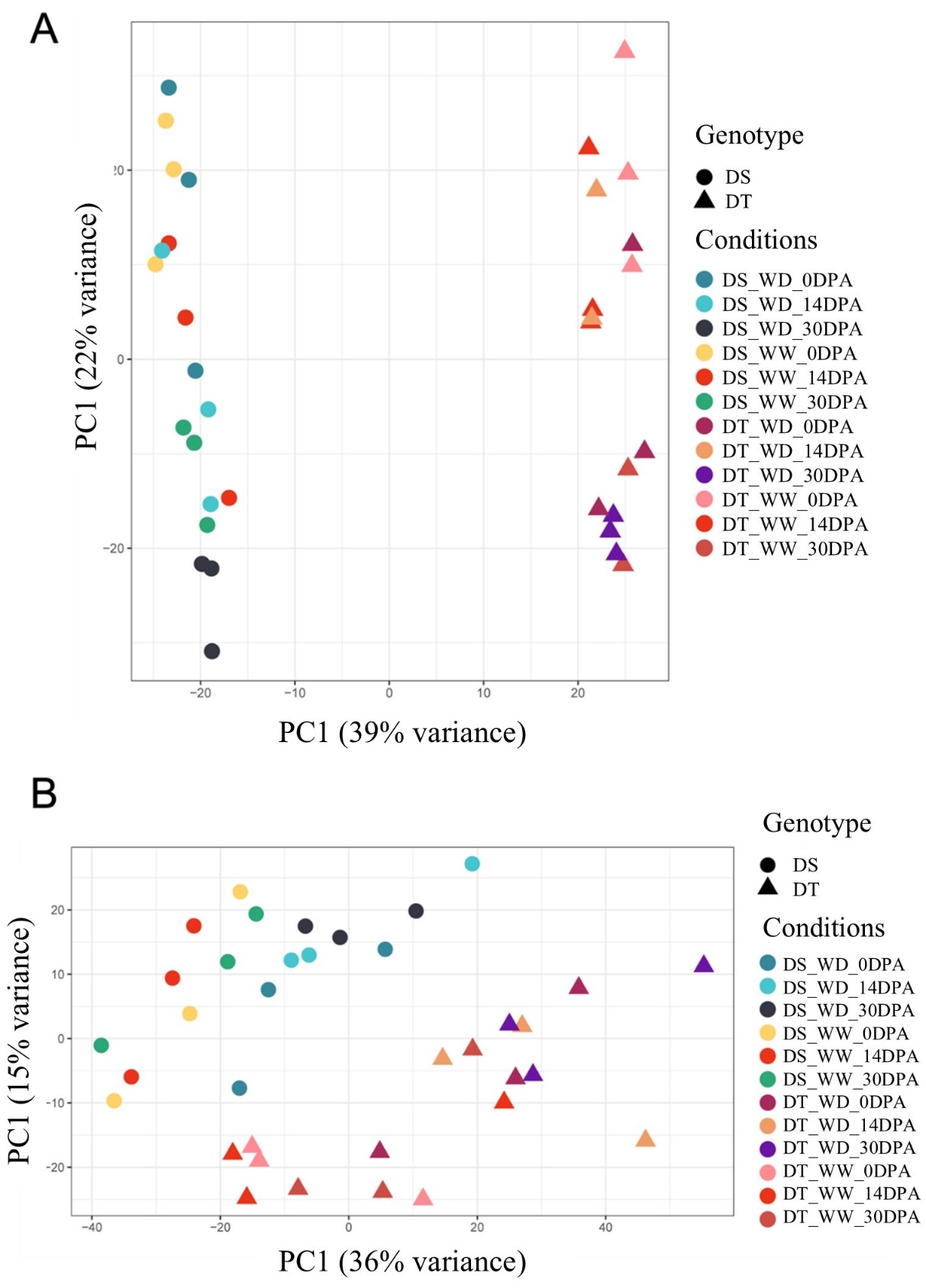


**Figure S7.** Principal component analysis using transcriptomics data in leaf (**A**) and root (**B**) samples. Abbreviations: DT - drought-tolerant genotypes; DS - drought-susceptible genotypes; WW - well-watered conditions; WD - water-deficient conditions; 0-, 14- and 30-DPA.


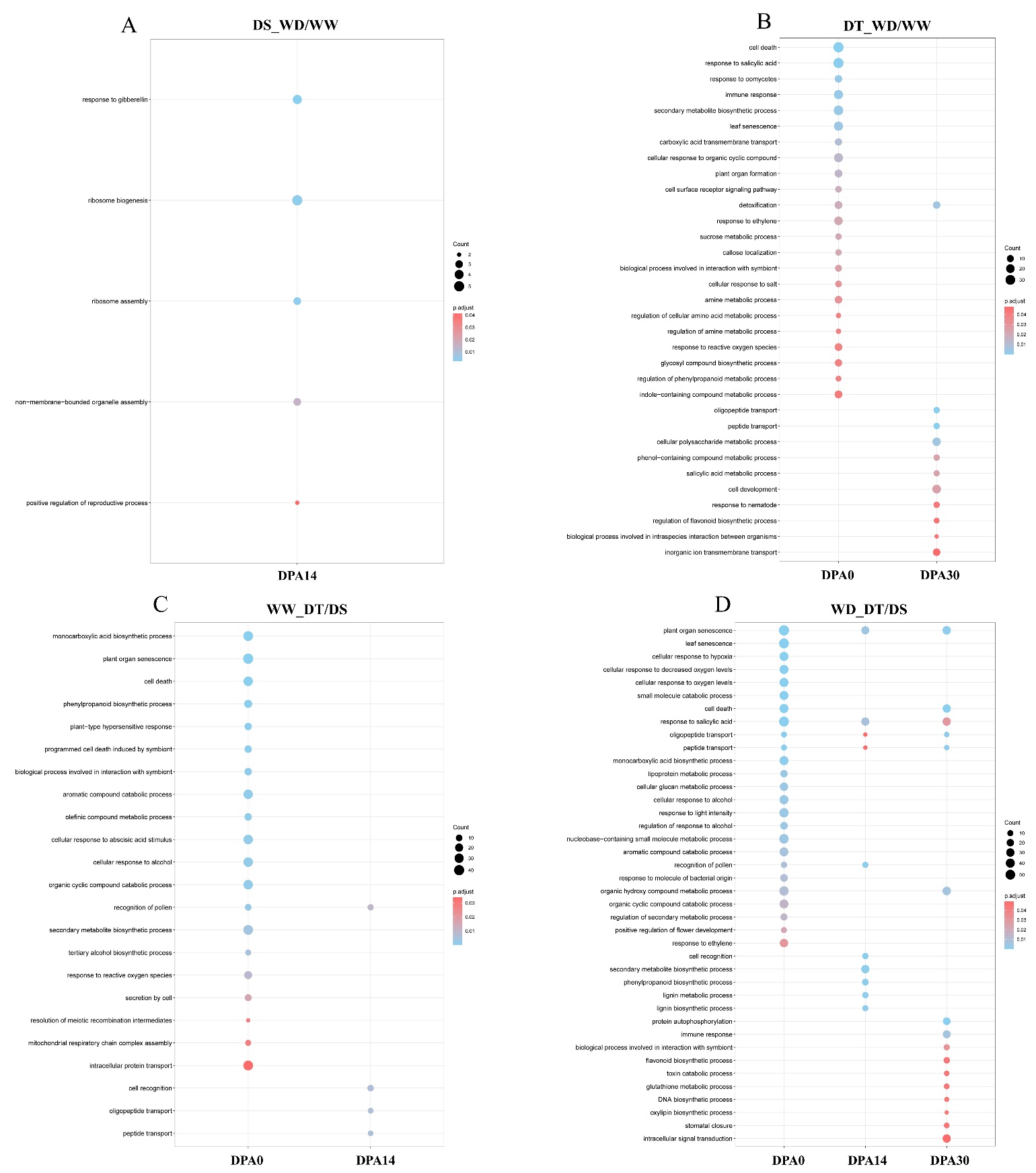


**Figure S8.** Gene Ontology (GO) enrichment analysis in the leaf by the comparison of WD/WW in the DS (**A**) and DT (**B**) genotypes, and DT/DS comparison under WW (**C**) and WD (**D**).


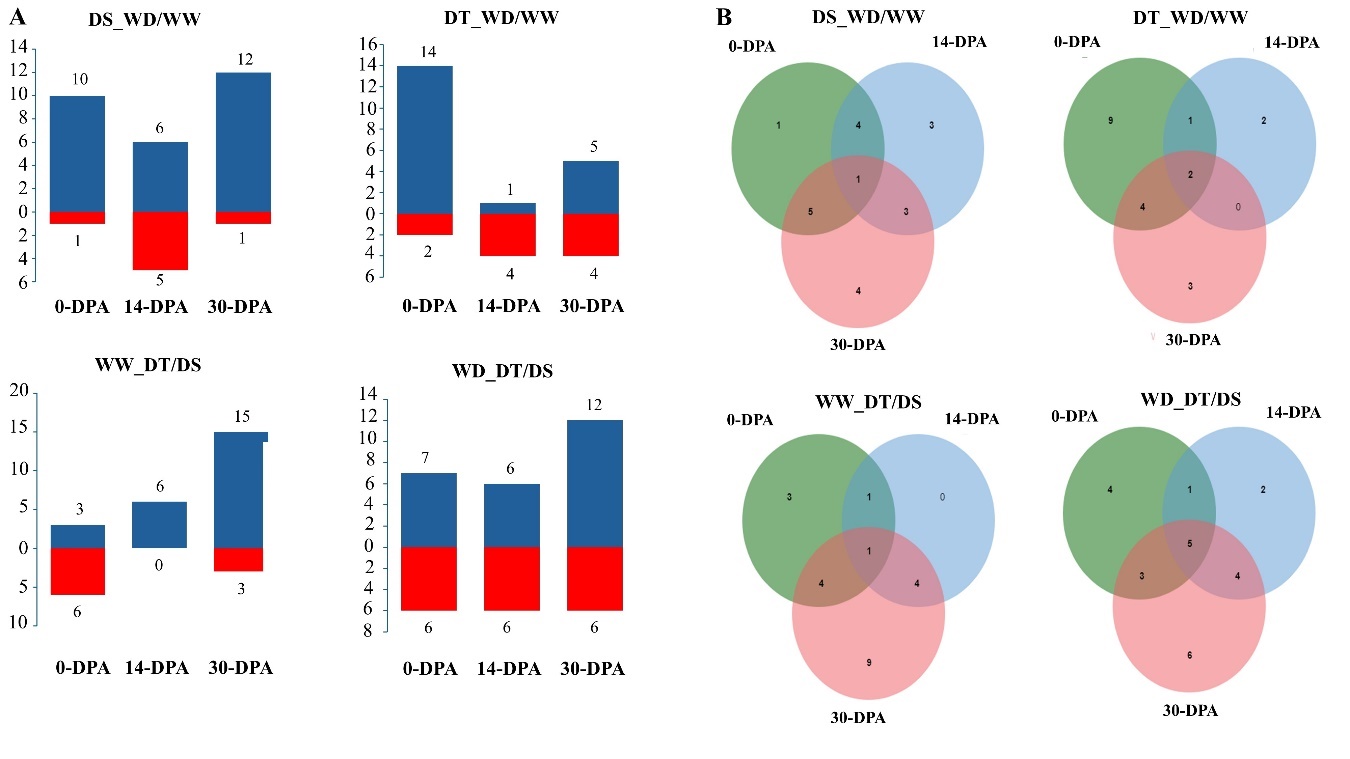


**Figure S9.** Total number of up- and down-concentrated metabolites identified by the comparison of WD/WW in the two genotypes and DT/DS under different water conditions at the three phenological stages (0-, 14- and 30-DPA) in leaf.


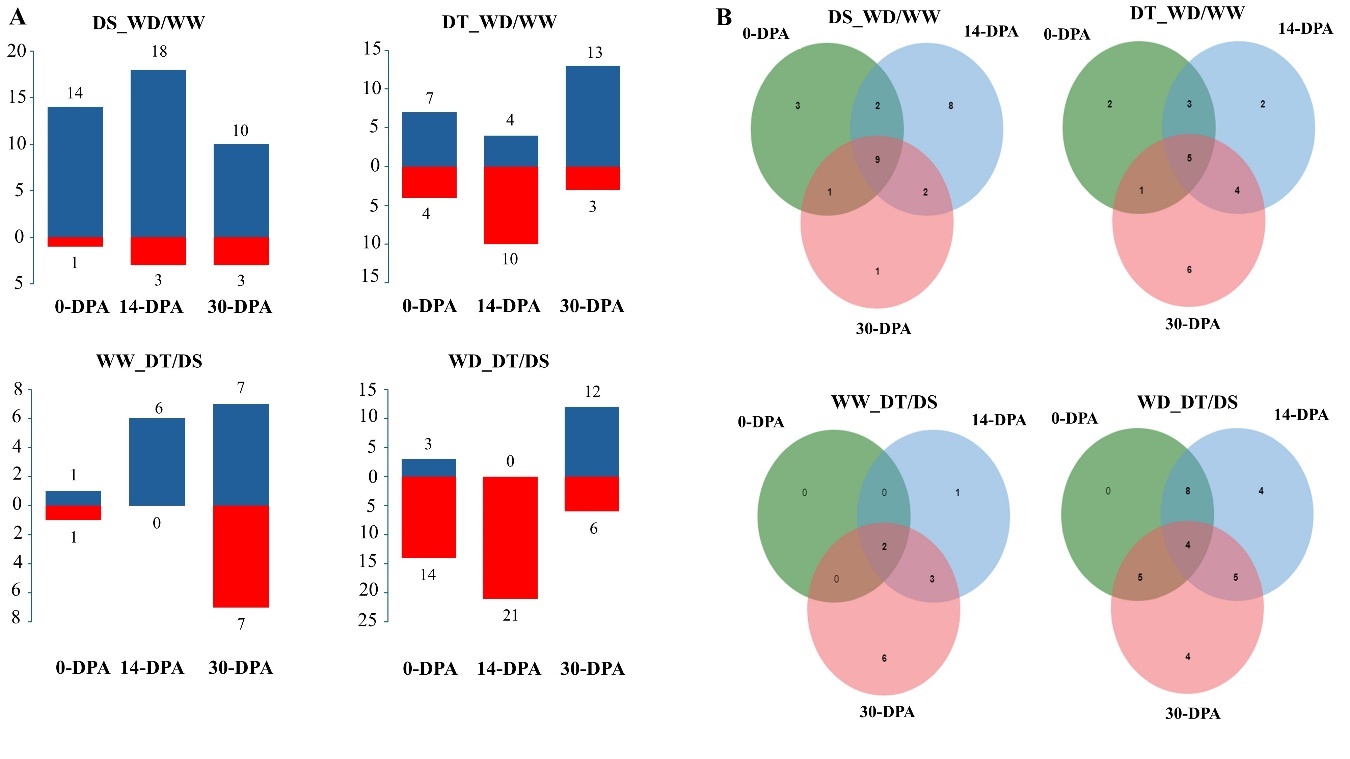


**Figure S10.** Total number of up- and down-concentrated metabolites identified by the comparison of WD/WW in the two genotypes and DT/DS under different water conditions at the three phenological stages (0-, 14- and 30-DPA) in root.
